# Targeting Lysosomes for Enhanced Anti-Cancer Therapeutics and Immune Response

**DOI:** 10.7150/ijbs.127929

**Published:** 2026-01-15

**Authors:** Michal Stark, Yehuda G. Assaraf

**Affiliations:** The Fred Wyszkowski Cancer Research Laboratory, Faculty of Biology, The Technion-Israel Institute of Technology, Haifa 3200003, Israel.

**Keywords:** lysosomes, LMP, autophagy, macrophage, immune response

## Abstract

Cancer is a leading cause of death in Western countries. Apart from surgical resection, the primary treatment modalities chemotherapy and radiotherapy inflict serious side effects, and significantly remodel both tumor metabolism and the tumor microenvironment. This consequently compromises treatment efficacy, resulting in multiple drug resistance, immune evasion and cancer progression. Lysosomes are unique acidic intracellular organelles crucial for maintaining cellular health and homeostasis via degradation of cellular waste. Lysosomes are also required for autophagy, a stress-induced catabolic pathway that is important for cell survival. Autophagy is typically enhanced in tumor cells, as it can confer cyto-protection against the deleterious cytotoxic effects of chemotherapy, and suppress anti-cancer immune response. Owing to their acidic nature and their role in endocytosis, lysosomes can be readily targeted and manipulated, thus attenuating the autophagic flux and improving cancer treatment outcome. Herein we focused on various classic and innovative lysosome modulators, their impact on autophagy, the enhancement of immune response, and consequent inhibition of tumor growth and metastasis. We discuss modalities to minimize adverse effects in cancer patients by either utilizing harmless compounds, achieving synergistic activity with combination therapies, or specifically targeting the tumor by using advanced nanoparticle technologies.

## Introduction

**Figure 1:**
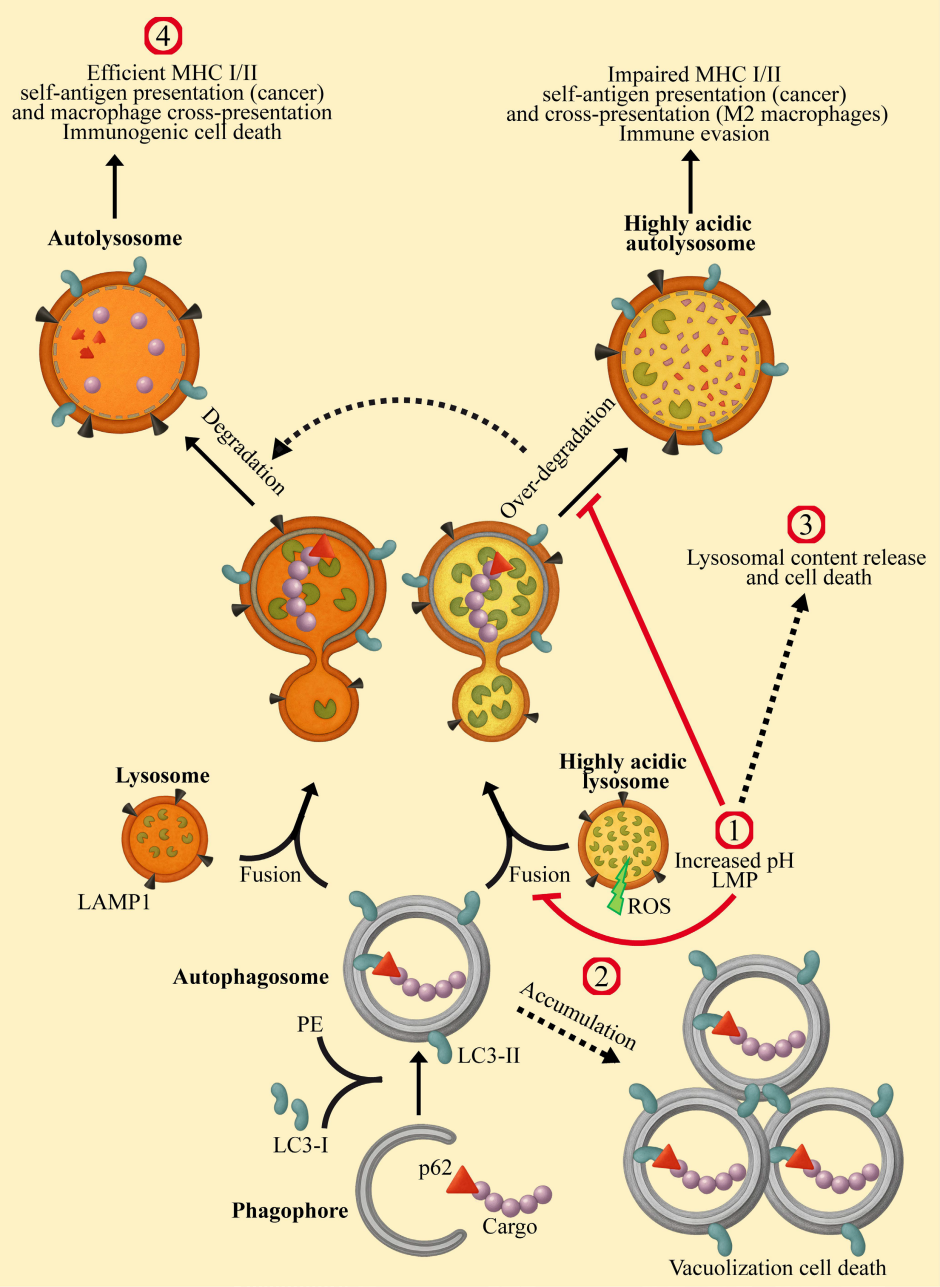
** Modulating the enhanced autophagy in cancer cells via lysosomal targeting.** The autophagy process is initiated by the formation of a cap shaped, double-membraned phagophore, which expands and elongates to capture cytoplasmic cargo. The cargo is tagged by ubiquitin-binding protein p62 (Sequestosome-1). Microtubule-associated protein 1 light chain 3 alpha (LC3) is recruited to the phagophore membrane through phosphatidylethanolamine (PE) lipidation to form a double-membrane autophagosome, decorated with LC3-II. This autophagosome fuses with a lysosome to form an autolysosome, in which the cargo and membrane proteins are degraded. The lysosomes of cancer cells and M2-macrophages are highly acidic, with increased activity of hydrolases. This results in over processing of the digested cargo, thereby preventing cancer antigen presentation, culminating in immune evasion. Lysosomotropic drugs (LDs) affect autophagy at the indicated sites: 1) Lysosomal alkalinization by CQ, HCQ, Naphplatin, hydrotalcite, TFP, and BCZT leads to LMP. Conversely, treatment with ginsenosides R_o_ and R_h2_, UIOQM-IQ, Lys05, and Ir-C_3_N_5_ induces LMP, resulting in lysosomal alkalinization. Both alkalinization and LMP can inhibit autophagosome-lysosome fusion and/or decrease degradation within autolysosomes, resulting in autophagy inhibition. 2) Inhibiting autophagosome-lysosome fusion results in the accumulation and enlargement of autophagosomes, leading to vacuolization cell death. 3) LMP mediates the release of lysosomal content to the cytosol, including ROS and hydrolytic enzymes, triggering mitochondrial depolarization and lysosomal cell death. 4) Lysosomal alkalinization decreases the activity of hydrolases, thereby reducing degradation of MHC and antigen molecules within autolysosomes. This potentiates immunity by enhancing self-antigen presentation on cancer cells and cross-presentation by macrophages.

Lysosomes are eukaryotic membrane-bound cellular organelles with an acidic lumen at a pH range of 4.5-5.5 1-4, containing approximately 60 hydrolytic enzymes 5 displaying optimal activity at acidic pH 6-8. These hydrolytic enzymes, including proteases, nucleases, lipases, glycosidases, phospholipases, phosphatases, and sulfatases, are responsible for the degradation of proteins, nucleic acids, lipids, glycosides, and cellular debris, including damaged organelles, thereby maintaining cellular health and homeostasis 9-12. Apart from their major degradative role, lysosomes are central sensory hubs which respond to multiple cues to regulate metabolism, cell differentiation and division, as well as apoptosis and tumorigenesis 13-16. Furthermore, lysosomes are key components and regulators of the homeostatic autophagy process 17-19. The latter involves the sequestration of damaged organelles, misfolded proteins, intracellular pathogens and other foreign substances in double-membrane vesicles known as autophagosomes, which fuse with lysosomes for cargo degradation and recycling (Figure 1) 20-22. Autophagy promotes either cell survival or cell death 23-27. As a stress-induced catabolic pathway, autophagy facilitates the adaptation of cells to stress conditions such as starvation, by breaking down damaged or non-essential cellular structures and macromolecules to provide essential metabolites 28,29. Mitochondrial damage triggers mitophagy (mitochondrial autophagy), by which it protects the cell against release of pro-apoptotic proteins and reactive oxygen species (ROS) generation 30. Endoplasmic reticulum (ER) stress, following accumulation of unfolded proteins can lead to ROS generation and cell death 31,32. Autophagy provides cyto-protection by degrading damaged ER, thereby attenuating ER-stress defense and apoptosis 33. Moreover, autophagy can induce anti-cancer drug resistance via several molecular mechanisms which were previously reviewed 34-36. Enhanced autophagy protects cancer cells from DNA damage 37-39, in part, by the timely degradation of proteins of the DNA damage repair (DDR) system, such as checkpoint kinase 1 (CHEK1) 40,41. This timely degradation prevents excessive retention of DDR proteins on damaged/repaired chromatin loci, allowing for their replacement by subsequent factors necessary for the next step in the DNA repair pathway. Hence, autophagy promotes enhanced DDR in cancer cells during treatment with DNA damaging modalities, such as 5-fluorouracil (5-FU) 42 and radiation therapy 43,44. Given the key role that autophagy plays in cancer development, progression and survival 35,45-48, concentrated efforts were focused on developing novel strategies for the inhibition of autophagy during cancer therapy 49-53. This development requires efficient assays for monitoring the autophagy steps (Figure 1), to identify and quantify autophagy inhibition 54,55.

Lysosomal targeting can be achieved using lysosomotropic drugs (LDs), primarily characterized by their hydrophobic weakly basic nature, which allows them to freely diffuse into cells and intracellular organelles 56-58. LDs diffuse in and out of the cellular membranous compartments, however, once they encounter the acidic lumen of the lysosome, they undergo protonation, become cationic and can no longer traverse the membrane. This results in their intercalation and accumulation within the lysosomes' membranes 59-61. This sequestration “sink” effect results in the accumulation of LDs in lysosomes at concentrations which are 1000-2500-fold higher than their extracellular drug concentrations within a few hours 62. From a mechanistic perspective, concentrated positively charged molecules at the water-interface of the membrane bilayer disrupt the electrostatic balance between the lipid headgroups, inducing repulsion between the choline groups of phospholipids and increasing the distance between neighbor lipids 59. This can lead to membrane fluidization 63-66 and lysosomal membrane permeabilization (LMP) 67. The utility of lysosomal protons (H^+^) for the protonation process and loss of protons following LMP, alkalinizes the lysosomal luminal pH 68,69. Lysosomal targeting can also be achieved by utilizing the endocytic pathway 70.

In this review we focus on strategies, from the last decade, for lysosomal targeting, leading to the induction of lysosomal dysfunction, LMP, lysosomal rupture and lysosomal cell death (LCD), with emphasis on autophagy inhibition, and immunogenic cell death (ICD). We discuss lysosomal targeting with single agents and in combination with other drugs (i.e., chemotherapeutics) or *bona fide* therapy modalities (i.e., radiotherapy and sonodynamic therapy) for the eradication of cancer cells and tumors. While targeting lysosomes with photosensitizers for efficient photodynamic therapy (PDT) is a rapidly growing research field, it has been widely reviewed in recent years 71-78 and hence will not be discussed herein.

## Targeting lysosomes for anti-tumor immune response

### Macrophage polarization

**Figure 2:**
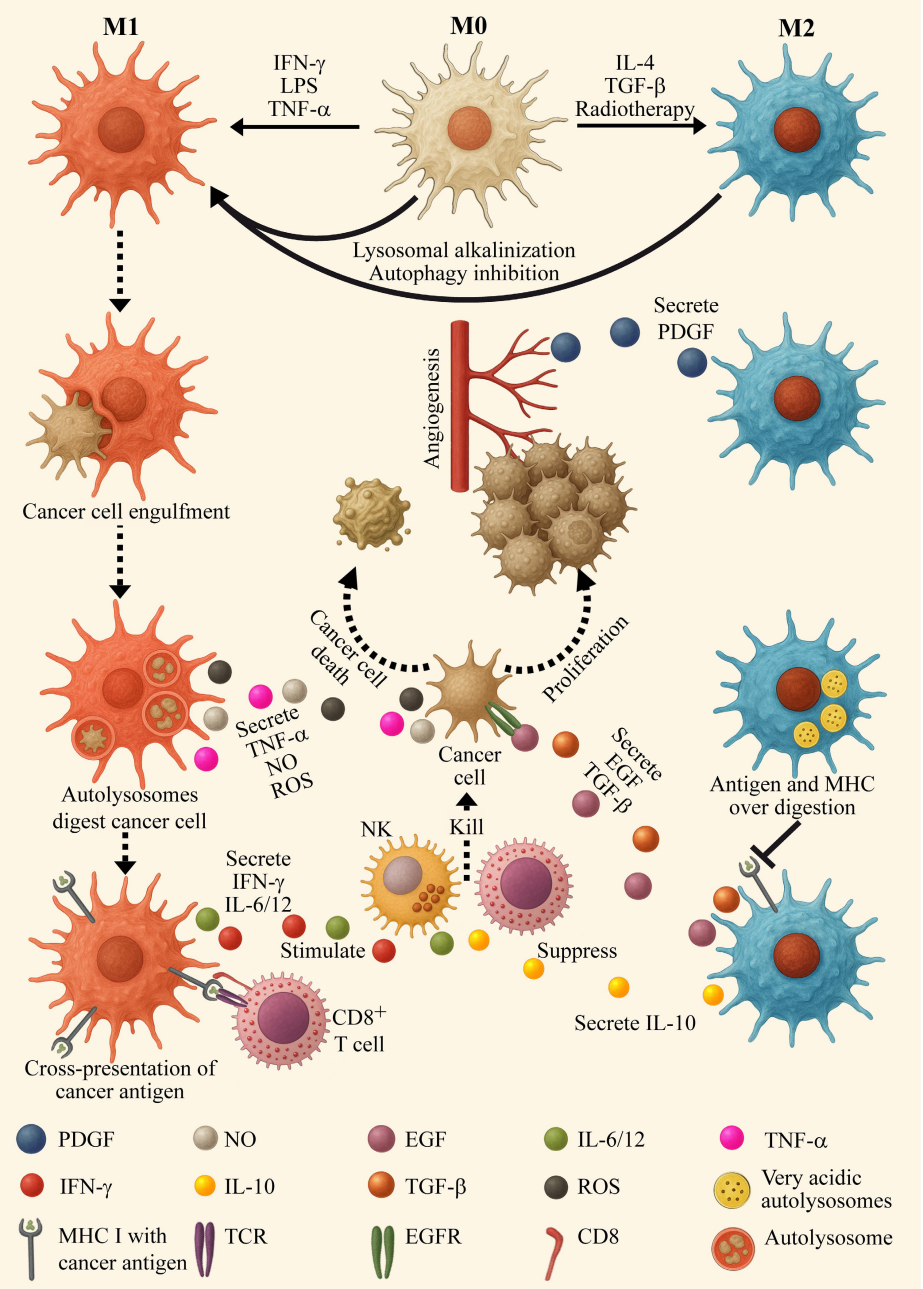
** Effectors of macrophage polarization and characteristics of the macrophage phenotypes.** On the left side, M0 resting macrophages undergo polarization to the pro-inflammatory/anti-tumoral M1 phenotype by microbial agonists of toll-like receptors, such as lipopolysaccharide (LPS), as well as the cytokines interferon gamma (IFN-γ) and tumor necrosis factor α (TNFα), which are then secreted by M1 macrophages themselves. The latter engulf and digest cancer cells via phagocytosis and autophagy. Lysosomes of M1 macrophages display relatively high pH for lysosomes (~5.3) and low hydrolase activity, resulting is moderate autophagy flux and cargo digestion. This results in optimal cancer-antigen sizes for cross presentation on MHC I, which then activates CD8^+^ cytotoxic T-cells. M1 macrophages secrete nitric oxide (NO), ROS and TNFα which elicit cancer cell death via apoptosis and necrosis. M1 macrophages activate and stimulate cytotoxic immune cells, including CD8^+^ T-cells and natural killer cells (NKs), by secreting pro-inflammatory interleukins including IL-6 and IL-12 as well as IFN-γ. On the right side: M0 resting macrophages undergo polarization to the anti-inflammatory/pro-tumoral M2 phenotype by naturally secreted IL-4 and transforming growth factor-β (TGF-β) as well as by radiotherapy (and other anti-cancer treatment modalities). M2 macrophages secrete pro-tumoral growth factors, such as platelet derived growth factor (PDGF), which promote angiogenesis, epidermal growth factor (EGF) and TGF-β that induce cancer cell proliferation, migration and invasion. M2 lysosomes present with high acidity (pH~4.5) and enhanced hydrolytic activity. This leads to the over-processing of cargos through the autophagy system, resulting in low cancer-antigen cross-presentation, facilitating immune evasion. M2 macrophages actively suppress the activity of CD8^+^ T-cells and NKs by secreting anti-inflammatory interleukins such as IL-10.

Macrophages are the predominant immune cell population present in cancer tissues 79,80. While macrophages have tumor-cell killing capacities, most experimental and clinical reports describe macrophages as protumor cells attenuating antitumor immune responses 81-84. Following macrophage polarization, they assume two main phenotypes designated M1 (pro-inflammatory) and M2 (anti-inflammatory) depicted in Figure 2 81-85. M1 macrophages are stimulated by interferon gamma (IFN-γ) and toll like receptor (TLR) ligands, including lipopolysaccharide (LPS) and lipoteichoic acid (LTA). Following stimulation, they express high levels of pro-inflammatory cytokines, including IL-1, IL-6, IL-12, IL-23, tumor necrosis factor α (TNFα) and IFN-γ, as well as inducible nitric oxide synthase (iNOS) 86. While M1 macrophages eliminate malignant cells, they also promote the antitumor cytotoxic activity of other leukocytes. Their enhanced tumor antigen presenting ability activates cytotoxic T-lymphocytes (CTLs, killer T cells, CD8^+^ T cells), and the cytokines they secrete stimulate and boost the function of natural killer (NK) cells.

In contrast, protumoral M2 macrophages act as immune suppressors 87. They have an impaired tumor antigen presentation ability, and secrete anti-inflammatory suppressor factors such as IL-10, hence negating any antitumor immune response. Furthermore, M2 macrophages release tumor promoting growth factors, such as platelet-derived growth factor (PDGF), transforming growth factor β (TGF-β) and vascular endothelial growth factor (VEGF), thereby encouraging tumor cell proliferation, angiogenesis and metastasis 81-85. Collectively, high prevalence of M2 tumor associated macrophages (TAMs) predicts dismal prognosis in various cancers, including lung 88, breast 89, pancreatic 90, and prostate cancer 91. It is well established that one of the most paramount characteristics that distinguishes between M1 and M2 macrophages is their lysosomal status. Lysosomes of M2 macrophages are more acidic than those of their M1 counterparts (pH ~4.5 vs ~5.3, respectively) 92-94. This affects the degradation process of biomolecules within the lysosomal lumen and the phagocytotic cascade, since many lysosomal proteases possess acidic pH optima below 4.5 6. M2 lysosomes display elevated hydrolase activity 95,96, leading to enhanced degradation of proteins (as well as lipids and nucleic acids), resulting in excessive antigen processing and diminished peptide presentation by MHC 97,98. This enhanced lysosomal activity also promotes an elevated autophagic flux in M2 macrophages 99,100.

In this respect, autophagy promotes lysosomal degradation of MHC I, thereby decreasing cancer-related antigen presentation, and facilitating immune evasion 101,102. Alkalinization of lysosomal pH with chloroquine (CQ) 101 or bafilomycin A1 (BafA1) 102 attenuated both the lysosomal enzymatic activity and the autophagic flux, resulting in higher surface presentation of MHC-I and an improved immune response against pancreatic cancer. This was also demonstrated by inhibiting the activity of the lysosomal protease cathepsin B (CTSB) 103.

Strategies are being developed to convert M2 to M1 macrophages, many of which utilize compounds that attenuate the lysosomal acidity and enzymatic activity. Naphplatin, a conjugation product of cisplatin to the core of the topoisomerase II inhibitor amonafide 104, was shown to localize in lysosomes of macrophages and increase their luminal pH 94. In turn, lysosomal alkalinization promoted the release of Ca^+2^ via the lysosomal cation channel mucolipin (Mcoln1), resulting in activation of the MAPK p38 signaling pathway. This led to the transformation of bone marrow derived macrophages (BMDMs) and M2-BMDMs to the pro-inflammatory M1-phenotype 94. In mice transplanted with murine colorectal cancer (CRC) CT-26 cells, treatment with cisplatin monotherapy induced an immune-suppressive response by increasing the percentage of tumoral M2 macrophages to 40% (*p* < 0.01). In contrast, naphplatin decreased the tumoral M2 macrophage population below 5%, while increasing the M1 population to ~80% (*p* < 0.01 and *p* < 0.001, respectively). This change was accompanied by a ~50% decrease in the levels of regulatory T cells (Tregs) in the tumor, and myeloid-derived suppressor cells (MDSCs) in the blood, further alleviating immune suppression. Naphplatin treatment boosted TAMs to inhibit CRC growth (final tumor weight was 10% of control) and pulmonary metastasis in CT-26 cells bearing mice, resulting in an 82.7% increase in survival time. To assess the contribution of macrophages to the anti-tumor effect of naphplatin, mice were also pretreated with the macrophage depleting agent clodronate 105, which abolishes mitochondrial ATP generation via inhibition of mitochondrial ADP/ATP translocase leading to apoptotic cell death. Pretreatment with clodronate diminished the anti-tumor effect of naphplatin, resulting in only 35% reduction in tumor weight. These findings establish the importance of lysosomal alkalinization to the immune response 94.

The same mechanism of increased lysosomal pH, consequent Ca^+2^ release and activation of MAPK p38 was demonstrated following treatment of mouse BMDMs and human macrophages with CQ 92. M2-TAMs were converted to the M1 phenotype, resulting in tumor growth inhibition and prolonged survival of B16 melanoma-bearing mice. Furthermore, following CQ treatment, mouse models of lung metastasis displayed a decreased number of tumor nodules in the lungs, and H22 hepatocarcinoma malignant ascites mouse models displayed reduced volume of ascites as well as reduced number of tumor cells 92.

pH-gated nanoparticles (PGNs) that self-assemble from amphiphilic copolymers were designed to accumulate within- and distinguish between lysosomes of M2-like BMDMs and other cell types, based on subtle lysosomal pH deviations 93. When conjugated to the TLR7/8 agonist imidazoquinoline (IQ), the now termed pH-gated nanoadjuvant (PGN_4.9_) selectively increased lysosomal pH in M2 macrophages, decreased cathepsin activity and converted these cells to the M1-phenotype. This in turn increased antigen presentation and activation of CTLs, resulting in tumor regression in a mouse 4T1 breast cancer model and further circumvented the formation of lung metastasis 93.

Yue Chen *et al.,* took a different approach by harnessing the increased expression/activity of CTSB in tumor cells 106-109 and M2 macrophages 95,110 to ignite a newly designed lysosomal “nanorocket” 111. The latter, termed UIOQM-IQ, consists of an ultrasmall iron oxide (UIO) nanoparticle (NP) conjugated to a CTSB-cleavable peptide, an aggregation-induced emission fluorophore QMTPA, and surface IQ. Following IQ-dependent endocytosis and internalization into lysosomes, CTSB cleaves the peptide within UIOQM, thus releasing QMTPA and UIO NPs with exposed -NH_2_ and -SH termini which drive cross-linking and aggregation of both QMTPA and UIO NPs. Both aggregates allow tumor visualization, with QMTPA activating a fluorescent “on” switch, and UIO inducing a distinct MRI contrast shift, enabling deep-tissue imaging. Most importantly however, the bulky UIO aggregates within lysosomes lead to elevated osmotic pressure, and consequent LMP 111. LMP can lead to lysosomal alkalinization and dysfunction, or to the release of lysosomal content into the cytosol, leading to LCD 112,113. The former will result in autophagy inhibition and macrophage polarization, and the latter should release cancer specific antigens and elicit an immune response. In fact, LMP is regarded as a mechanism driving ICD which triggers an intact antigen-specific adaptive immune response 114,115. Murine mammary carcinoma 4T1 cells express higher levels of CTSB than normal 3T3 murine fibroblast cells 111,116, hence UIOQM-IQ elicited a specific cytotoxic effect on 4T1 but not on 3T3 cells (surviving fractions ~20% and > 90%, respectively) 111. *In vivo* evaluation of UIOQM-IQ was conducted using 4T1 cells bearing mice. The lysosomal “nanorocket” promoted a robust anti-tumor immune response, represented by a remarkable increase in M1:M2 macrophage ratio from ~2 to > 30 (*p* < 0.0001), > 4-fold increase in secretion of TNFα and IFN-γ as well as IL-6 and IL-12. Moreover, an increase in the percentage of mature dendritic cells in tumor-draining lymph nodes (TDLN), and an increase in infiltrating CTLs was also noted, along with a decrease in the levels of Tregs. The combination of the anti-tumor immune response and the cytotoxic effect of UIOQM-IQ resulted in 5-fold smaller tumors and longer survival time (~35 days vs. > 60 days for control and UIOQM-IQ treated mice, respectively). Notably, UIOQM-IQ reduced lung metastases by ~8-fold 111.

**Figure 3:**
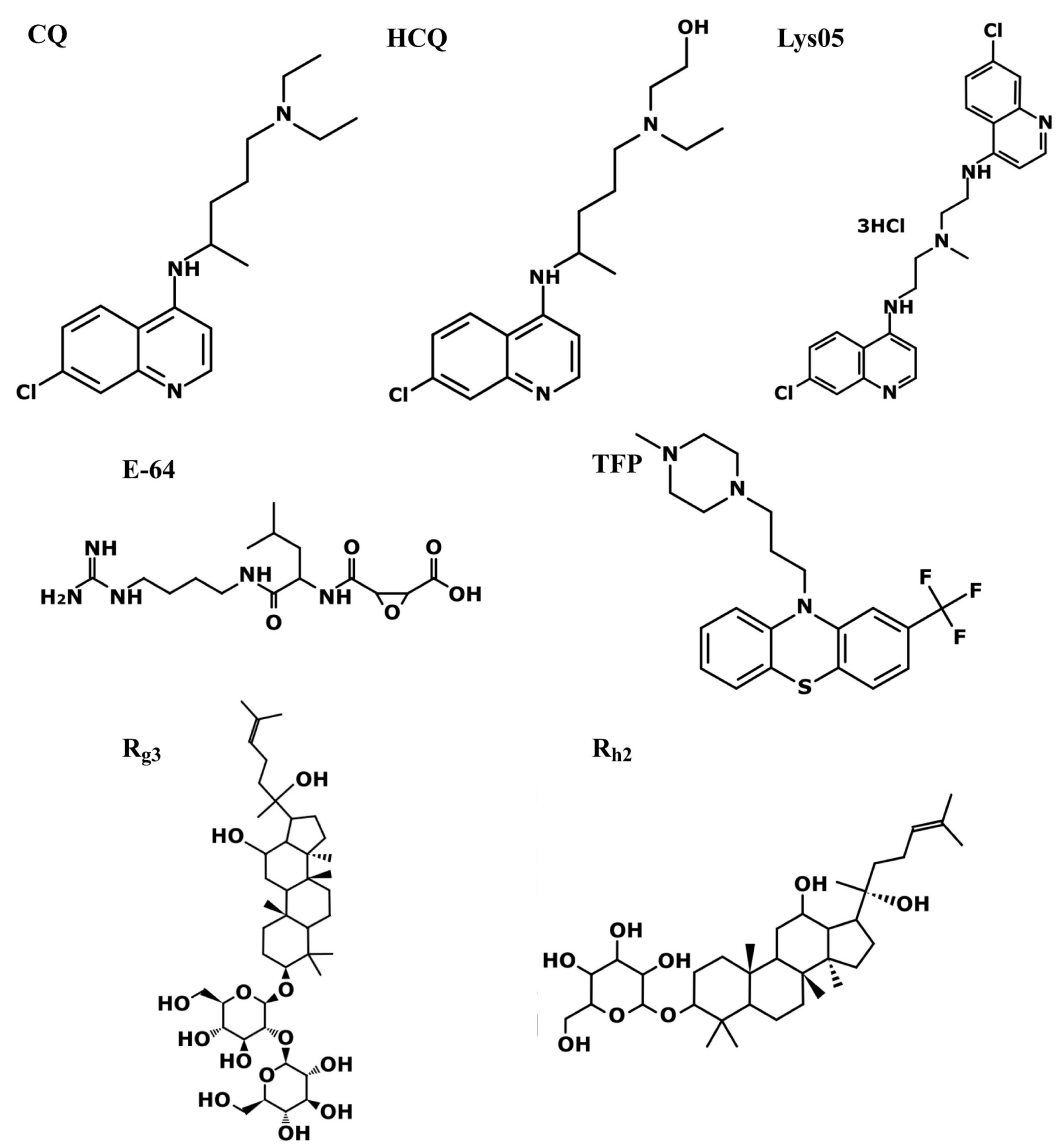
** Structures of various LDs described in the current review.** The anti-malarial drug chloroquine (CQ), its derivative hydroxychloroquine (HCQ), and its bisaminoquinoline dimeric form Lys05. The irreversible and selective cysteine protease inhibitor E-64. The antipsychotic phenothiazine trifluoperazine (TFP). One of the primary pharmacologically active constituents of ginseng, ginsenoside R_g3_ and its deglycosylated derivative R_h2_. Excluding ginsenosides, all molecules have hydrophobic rings and a hydrophilic amine group which undergoes protonation in acidic lysosomes, leading to their lysosomal accumulation, where they elicit lysosomal alkaliniziation and/or LMP. The ginsenosides act by triggering LMP by increasing cytosolic ROS levels. The molecules were generated using PubChem Sketcher V2.4.

E64-DNA, a DNA nanodevice composed of the small-molecule cysteine protease inhibitor, E-64 (Figure 3) 117, conjugated to a 38-base pair DNA duplex, was developed to selectively enter TAMs via endocytosis and accumulate within their lysosomes. Once there, E64-DNA inhibited lysosomal-specific cysteine proteases which are elevated in M2-TAMs, thereby increasing the cells' ability for antigen cross- presentation and effective CTL activation 95. When E64-DNA was combined with the widely used alkylating chemotherapeutic cyclophosphamide 118, sustained tumor regression was achieved in a triple negative breast cancer (TNBC) mouse model 95.

### *Panax ginseng* ginsenosides

In an effort to potentiate anti-tumor immunity with minimal chemotherapy-inflicted adverse effects, as well as to improve the quality of life of cancer patients, strategies are being developed for the combined treatment of herbal agents along with chemotherapy 119-122. One of the most commonly used roots that has been subjected to extensive anti-cancer research is ginseng, primarily its pharmacologically active constituents ginsenosides 123-127.

Ginsenosides are triterpene saponins (Figure 3), which include major ginsenosides and their secondary metabolic derivatives 128, of which R_g3_ 129,130 and its deglycosylated derivative R_h2_ 131,132, exhibit the most beneficial biological activities in various human pathologies. Regarding the present review, various ginsenosides were shown to increase lysosomal pH 42,133, induce LMP 133,134, inhibit autophagy flux 42,133,135-138, and enhance immunity 126,139-141. These resulted in sensitization to chemotherapy/immunotherapy 42,135,137-139,142,143, and most importantly enhanced *in vivo* anti-tumor activity 136,139,141,143,144.

It should be emphasized that although ginsenosides affect lysosomal function, they are not LDs according to their physicochemical properties, endowing them with both low water solubility and poor membrane permeability 145. Ginsenosides R_o_ 146,147 and R_h2_ were shown to increase cytosolic ROS levels in cancer cells, either via the estrogen receptor 2 (ESR2)-neutrophil cytosolic factor 1 (NCF1)-ROS axis 42, or mitochondrial ROS production 133. Extralysosomal ROS can damage the lysosomal membrane, and induce LMP with consequent lysosomal alkalinization, as was demonstrated upon mitochondrial ROS generation 148,149. This was also the case with R_o_ and R_h2_ 42,133,134. As abovementioned, lysosomal alkalinization reduces the activity of resident hydrolases; consistently, treatment with R_o_ reduced the activity of CTSB and CTSD 42. Lysosomal alkalinization can also inhibit autophagosome-lysosome fusion 150. Both decreased lysosomal enzymatic activity and autophagosome-lysosome fusion result in a reduction in the autophagic flux, as was observed following R_o_ or R_h2_ treatment 42,133. The ability of ginsenoside R_h2_ to inhibit autophagy was utilized to reverse the phenotype of RAW264.7 derived M2 macrophages to the M1 subtype *in vitro* 140. While an R_h2_ liposome (R_h2_-lipo), where R_h2_ functioned both as a cholesterol substitute membrane stabilizer, and a chemotherapy adjuvant, successfully polarized macrophages to the M1 phenotype *in vivo* 141. The increase in M1 macrophages in an orthotopic breast cancer 4T1 tumor bearing mouse model injected with R_h2_-lipo, was accompanied by residual levels of IL-10 and consequently high levels of activated CTLs and a dramatic decline in Tregs. The resulting potentiation of the immune response led to a 40% decrease in tumor volume, an impact which was enhanced to 80% via the co-encapsulation of R_h2_ with paclitaxel (PTX) 141. Utilizing R_h2_ as a component of the liposome membrane allowed tumor targeting via the glucose transporter 1 (GLUT1) which recognizes ginsenosides as substrates 151, and is overexpressed in tumors 152.

Ginsenoside R_g3_, another late-stage autophagy inhibitor 137,138, was also incorporated as a cholesterol substitute in the membrane of liposomes (R_g3_-LPs) 139. In this study, the affinity of ginsenoside-liposomes for GLUT1 was exploited to both penetrate the blood-brain-barrier (BBB), and deliver PTX for the targeted eradication of C6 glioma brain tumors in both mice and rat models. R_g3_-LPs, and to a greater extent R_g3_-PTX-LPs, polarized M2 macrophages to the M1 phenotype, both *in vitro* and *in vivo*, along with an 80% decline in the levels of TGF-β. The immune activation in the tumor microenvironment (TME) drastically reduced the presence of MDSCs and Tregs, while increased the abundance of CTLs from 5% to 30-40%. Collectively, these favorable activities dramatically increased the survival of C6 glioma bearing mice from 21 days to 32 and 54 days for R_g3_-LPs and R_g3_-PTX-LPs, respectively (*p* < 0.01). In the rat model, the survival was even longer, with the R_g3_-PTX-LPs group exceeding the experiment time of 60 days 139.

A systematic review analyzed the impact of combining first-line chemotherapy with the ginsenoside R_g3_ in the treatment of advanced non-small cell lung cancer (NSCLC) 153. Analysis of 2,200 NSCLC patients from China revealed excellent results highlighting the beneficial effects of R_g3_ for cancer patients. When compared to the control group receiving first-line chemotherapy alone, the R_g3_ supplemented patients exhibited higher response rates (*p* < 0.00001), higher Karnofsky performance status index (*p* < 0.00001), higher one- and two-year survival rates (*p* = 0.01, *p* = 0.006), higher rate of weight improvement (*p* = 0.02), reduced VEGF levels (*p* = 0.02), less gastrointestinal adverse effects (*p* = 0.02) as well as lower rates of myelosuppression (*p* < 0.00001) 153. An additional review analyzed the benefits of ginsenosides R_h2_, R_g3_ and compound K 154,155 as adjuvant therapy in 1,448 hepatocellular carcinoma patients, demonstrating similar remarkable results 156. It should be conveyed, that since ginsenosides display pleiotropic activities 129-132, the beneficial effects they elicit as adjuvants cannot be solely attributed to their lysosomotropic effects.

## Radiotherapy

Radiation therapy *aka* radiotherapy (RDT) is an established hallmark of cancer treatment, along with chemotherapy, immunotherapy, hormone therapy, and surgery 157-159. RDT employs high-energy ionizing radiation in order to elicit two major antitumor activities 160,161. The first is the obvious eradication of the irradiated target cells by inducing DNA damage, mitotic catastrophe and apoptosis 162. The second, is referred to as *in situ* tumor vaccination 163,164 which stimulates a systemic immune-mediated antitumor response. Following RDT-induced cell death, the local release of tumor-derived antigens promotes their cross-presentation by various antigen presenting cells (APCs), which in turn instigates an immediate and prolonged immune response via the activation of NK cells, CTLs and B-cells 165-168. This “vaccination” can elicit an abscopal effect, where irradiation of a small tumor area induces a systemic antitumor immune response throughout the body, resulting in regression of tumors in remote, untreated parts of the body 169-171. Inasmuch as this sounds promising, RDT is actually effective in only a fraction of cancer cases, while in others it has the exact opposite effect. In this respect, various studies have shown that following RDT, a burst in immune-suppressive stimuli occurs 162, leading amongst others, to an elevation in pro-tumoral M2 macrophages 162,172,173. Moreover, using the lung colonization model of transplanted murine 4T1 breast cancer cells, RDT was shown to enhance lung metastasis in mice 173,174, and the pro-metastatic impact required the presence of macrophages 174. Although studies have shown that lysosomes and autophagy are main contributors to this RDT-resistance 159,175,176, only a few studies have attempted to target lysosomes to overcome this radioresistance.

A very recent study that targeted lysosomes, followed the example of successfully enhancing immunotherapy by alkalinizing the lysosomes of macrophages, and implemented this strategy following RDT treatment 173. Using the mouse 4T1 orthotopic breast cancer model, Bei Li *et al.,* demonstrated that an immune-suppressive TME was established following RDT. This included a high prevalence of M2 macrophages and poor tumor infiltration of CTLs 173. Post-RDT macrophages were stimulated with cytidine monophosphate guanosine oligodeoxynucleotide (CpG), a TLR 9 agonist, resulting in the rewiring of their central carbon metabolism. This promoted these macrophages to engulf and eradicate tumor cells for antigen cross-presentation 177. However, since M2 macrophages have extremely acidic lysosomes with enhanced hydrolytic activity, as mentioned above, the antigens were over-processed, abolishing antigen-presentation by MHC I. Thus, the authors administered a MgAl-based hydrotalcite (bLDH) alkaline nanoadjuvant to the peritumoral area post-RDT. Hydrotalcite is an antacid currently used to neutralize stomach acidity 178,179. While the combination of CpG and bLDH was sufficient to increase surface localization of antigen presenting MHC I in macrophages, the effect was even stronger post RDT. A previous study with hydrotalcite-embedded magnetite NPs, showed the accumulation of these NPs in lysosomes 180. Consistently, bLDH induced lysosomal alkalinization in BMDMs. The enhanced cross-presentation following co-administration of CpG and bLDH resulted in priming of antigen-specific CTLs and tumor infiltrating NKs leading to the consequent suppression of the primary tumor and lung metastasis in the mouse 4T1 breast tumor model 173.

The brain tumor glioblastoma multiforme (GBM) is a highly aggressive and fulminant malignancy which displays immune-evasion 181, chemoresistance and radioresistance 182,183. In the latter respect, to surmount this RDT resistance, Xin Zhang *et al.,* utilized trifluoperazine (TFP, Figure 3) 184, an antipsychotic phenothiazine from the 1950s 185. TFP has been shown to inhibit proliferation, migration, and invasion of GBM cells, however it failed to extend the survival time of orthotopic U87MG xenograft bearing mice 186. In contrast, when combined with RDT, TFP significantly increased the survival of orthotopic xenograft GBM mouse models with P3 cells (median survival 46.0 vs 29.7 days for combined treatment vs. radiation alone, *p* < 0.01) 184. TFP is a highly hydrophobic weak base compound 187, like the majority of anti-psychotic drugs, and thus highly accumulates in lysosomes 59,188. Indeed, Xin Zhang *et al.,* demonstrated lysosomal alkalinization following treatment with TFP, along with a decrease in the activity of lysosomal cathepsin proteases, and a consequent decrease in the autophagic flux 184. In a follow up paper, this group further revealed that TFP induced lysosomal swelling and LMP, resulting in reduction of the autophagic flux 189. However, the radio-sensitization achieved by TFP was attributed to impaired homologous recombination during radiation-induced DDR, with no mention of abrogating RDT-induced immune-suppressive responses 184.

Qi Xu *et al.,* developed a core-shell copper selenide-coated gold (Au@Cu_2-x_Se) NPs which were shown to minimally affect lysosomal pH, and to prominently block the autophagic flux 190. This resulted in radio-sensitization and tumor eradication, leading to prolonged survival of orthotopic mouse xenograft GBM model harboring human U-87MG cells (median survival 42 vs 29 days, for combined Au@CS + X-Ray treatment vs. X-Ray alone) 190. These NPs required focused ultrasound (US) to better traverse the BBB and reach the intracranial tumor site.

### Chloroquine

The anti-malarial drug CQ (Figure 3) has been widely studied in the treatment of various pathological disorders 191 including cancer 192,193. Its lysosomotropic properties have been exploited for lysosome alkalinization (as discussed in the immunotherapy chapter) and autophagy inhibition 194. These properties have also been exploited to sensitize tumors to RDT, primarily GBM.

Glioma initiating cells (GICs) 195 are radio-chemo-resistant stem-like cells responsible for relapse following treatment of GBM with RDT 196,197. Early studies demonstrated that enhanced autophagy promotes differentiation of GICs 198, decreases their tumorigenicity 199 and restores their radio-sensitivity 200. In contrast, in recent years, autophagy inhibitors were shown to increase sensitivity of GICs to both RDT 201 and chemotherapy 202. Chenguang Li *et al.,* settled the dispute by showing that inhibition of autophagy at different stages of the process has distinct effects 203. Blocking the autophagic flux at the end of the process, i.e., autophagosome-lysosome fusion and/or content degradation, leads to the accumulation of degradative vacuoles, and resensitizes GBM cells to anti-cancer treatments.

Autophagy inhibition by CQ at the stage of autophagosome-lysosome fusion 194 potentiated the radio-sensitivity of GICs isolated from the human glioma cell line U87 201. The combination of CQ and X-ray markedly decreased the clonogenic surviving fraction of GICs by ~10-fold compared to X-ray alone, and increased apoptosis by a factor of > 2-fold. Finally, the combination exhibited a synergistic activity on GICs generated tumor spheres, decreasing both their numbers and diameters 201.

The potential of CQ in restoring radio-sensitivity to GBM has been tested in several clinical trials over the past 20 years with encouraging outcomes (Table S1) 204-209. When presenting increased progression free survival (PFS) and median survival time after surgery for CQ-treated patients, it emerged that these clinical trials could have been the initiators of routine CQ administration in GBM treatment. However, the patient numbers in all these trials were too small to attain statistical significance and draw definitive conclusions. For example, the Sotelo group published the results of a prospective controlled randomized trial, where nine patients receiving an additional daily dose of 150-mg CQ to the radiochemotherapy, were compared to nine control patients. The mean survival time was 31±5 and 10.6±2 months, respectively,* p*<0.0002 204. Later, the same group published a post-surgery median survival time of 24 months for CQ-treated patients (n=15) and 11 months for control patients (n=15), with double the number of survivors in the CQ-treated patients at the end of observation (*p* = 0.139) 205. Furthermore, a study on the treatment of recurrent GBM (rGBM), retrospectively compared 33 patients in a control group receiving only adjuvant-radiochemotherapy (aRCT) to those receiving aRCT+ bevacizumab (BEV, n = 5) or the triple combination: aRCT+BEV+CQ (n = 4). Median post recurrence survival times were 9.63, 12.97 and 23.92 months, respectively, *p* = 0.022 209.

Some beneficial effects were clinically attributed to the addition of CQ in combination with standard chemotherapy, in case of advanced or metastatic anthracycline-refractory breast cancer 210 and metastatic or unresectable pancreatic cancer 211 (Table S1). The maximal tolerable CQ dose in clinical trials was found to be 200-250 mg/day 208,209,212. Higher doses of CQ elicited multiple adverse effects, including irreversible blurred vision and vomiting 208. Although CQ is an excellent lysosomal alkalinizing agent, its tolerable dose might not be sufficient to allow for effective autophagy inhibition required for chemo/radio/immuno-sensitization. One plausible modality to circumvent these adverse effects of high dose CQ is its encapsulation and tumor targeting 213.

### CQ encapsulation

Temozolomide (TMZ), a DNA alkylating and methylating agent, is the first-line chemotherapeutic drug in the treatment of GBM and anaplastic astrocytoma 214,215. However, like most cytotoxic drugs, TMZ inflicts adverse effects with up to 20% of glioma patients suffering from thrombocytopenia 216. The combination of TMZ and CQ has been shown to bear a synergistic effect in eradicating GBM cells 217,218, primarily via lysosomal dysfunction and autophagy modulation 218. Furthermore, this combination displayed promising results in clinical trials 205,208. Hence, the combined encapsulation of these drugs in targeted NPs has the potential to exert synergistic anti-tumor effects without harming healthy tissues.

Mesoporous silica NPs (MSNs) have been extensively used experimentally for drug delivery *in vivo* 219. The incorporation of polydopamine (PDA) into MSNs allows to modify the surface of the NPs and attach specific ligands for cancer targeting 220. PDA also adds a pH-responsive element to the NPs as it undergoes degradation under acidic conditions, enhancing drug release in lysosomes or in the acidic TME 221,222. The arginyl-glycyl-aspartic acid (RGD) tripeptide 223,224 is an established ligand of α_v_β_3_ integrin used for *in vivo* tumor mapping and targeting 225-227. The surface integrin α_v_β_3_ is crucial for tumor angiogenesis and is highly expressed predominantly in new blood vessels 228,229, as well as various tumors 230-234 including GBM 230,235. TMZ and CQ were co-loaded into MSNs coated with PDA decorated with RGD, designated TMZ/CQ@MSN-RGD 236. *In vitro* growth inhibition assays, using the α_v_β_3_ integrin expressing human GBM cell line U87 237, showed a 4-fold lower IC_50_ value for TMZ/CQ@MSN-RGD compared to free TMZ (24.5 vs. 104.3 µg/ml, respectively), while TMZ/CQ@MSN-RGD had little effect (< 15%) on the viability of rat cortical neuronal cells, even at a high concentration of 1 mg/ml. These results suggest the specificity of the NPs for GBM cells. By accumulating in lysosomes (following endocytosis), inhibiting autophagy, and enhancing apoptosis, TMZ/CQ@MSN-RGD inhibited tumor growth in U87 cells xenograft bearing mice, twice as much as free TMZ, with no apparent toxicity to healthy organs 236.

A cyclic RGD peptide was also used to coat lipid NPs (LNPs) co-loaded with CQ and the anti-malarial drug dihydroartemisinin (DHA) 238. Apart from integrin α_v_β_3_ being a target, RGD is also an established ligand of integrin α_v_β_6_ 224,239,240, which is highly overexpressed in CRC, where it enhances tumor aggressiveness 241-244. DHA is considered a sensitizing agent shown to increase ROS levels in cancer cells 245-247, however, its pharmacologic effect as a single agent is usually abrogated by the activation of protective autophagy 248-250. Hence, it was determined that DHA should be combined with other cytotoxic agents 251, preferably an autophagy inhibitor 252,253. This was the rational for the design of LNPs co-loaded with CQ and DHA and decorated with RGD (RLNP/DC) for the treatment of CRC 238. Even when loaded with a relatively low CQ concentration of 7.5 µM, RLNP/DC exhibited superior potency in inhibiting colony formation, increasing ROS production, inhibiting autophagy, and increasing apoptosis in CRC HCT116 cells compared to free CQ+DHA. The results obtained from *in vitro* invasion and migration assays suggested an anti-metastatic potential for RLNP/DC. Consistently *in vivo*, murine HCT116 cell models of CRC and metastasis, presented with ~3-fold less CRC tumors, which were ~3-fold smaller upon treatment with RLNP/DC, compared to free CQ+DHA. RLNP/DC-treated mice also developed half the number of liver metastases than those treated with free drugs, and exhibited a 25% increase in survival rates, with all mice being alive at the end of the 60 days experiment. RLNP/DC-treated mice also maintained the highest body weight throughout the experiment 238.

### Encapsulation of hydroxychloroquine

**Figure 4:**
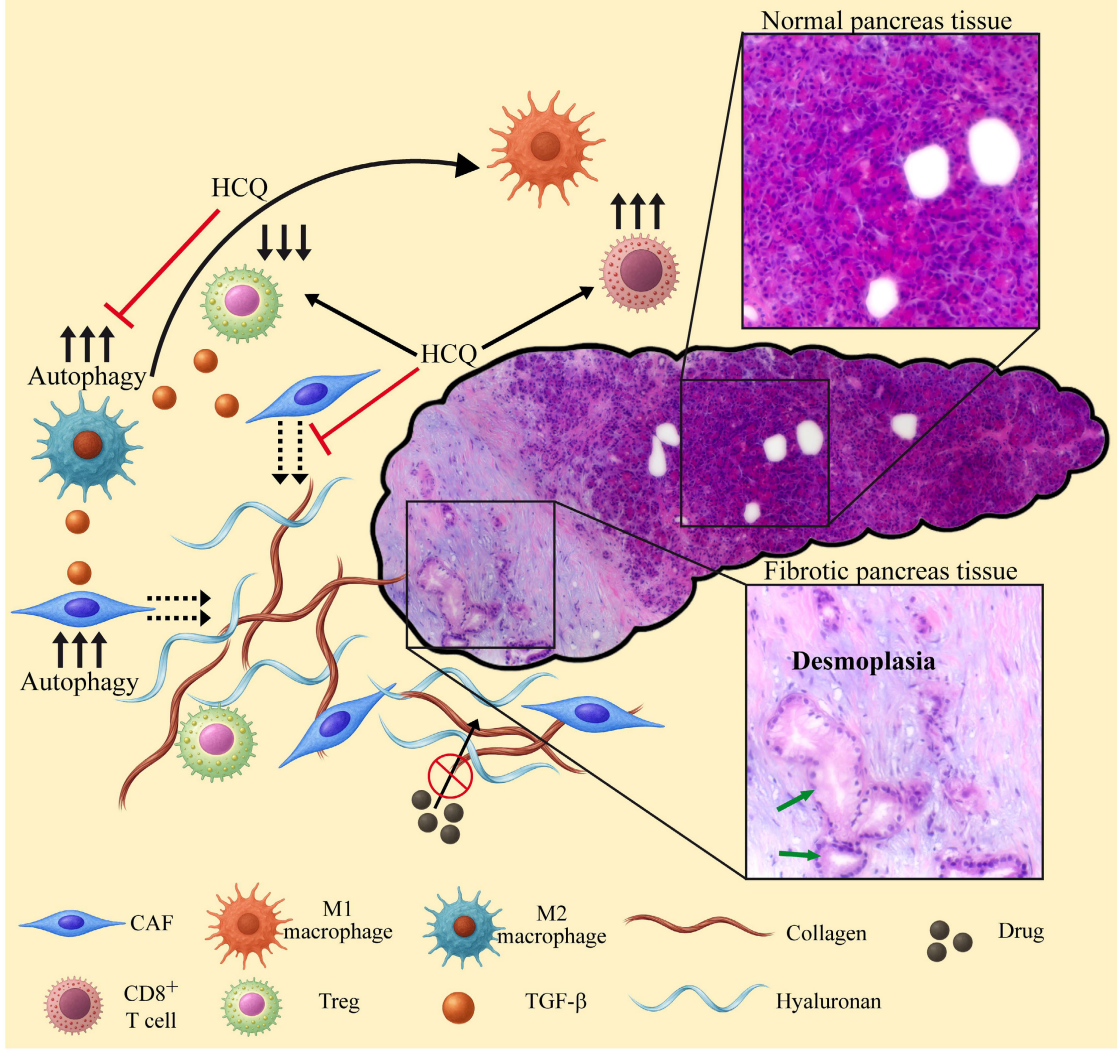
** The role of autophagy and inhibition of autophagy in reprograming the TME and TIME of PDAC.** High levels of autophagy in cancer associated fibroblasts (CAFs) promote the secretion of ECM components including collagen and hyaluronan. High levels of autophagy in macrophages result in an M2 phenotype, leading to the secretion of TGF-β. The latter further increases autophagy in TME cells, enhances ECM stiffness and stimulates cancer cell proliferation. The dense desmoplastic barrier prevents accessibility of various anti-cancer drugs to the tumor cells. M2 macrophages suppress anti-tumor immune responses, stimulating the differentiation of Tregs that further suppress the immune system. Lysosomotropic drugs like HCQ, block autophagy, thereby reprogramming the TME and TIME as follows: 1) M2 macrophages undergo polarization to the M1 phenotype, thus stimulating the activity of CTLs, reducing the levels of Tregs and TGF-β. 2) The secretion of collagen and hyaluronan by CAFs is halted, thereby preventing the biosynthesis of new ECM. Consequently, the desmoplastic barrier is relieved, restoring the accessibility of anti-cancer drugs to the tumor cells as well as the infiltration of active immune cells. The normal and fibrotic pancreatic tissues shown are representative hematoxylin and eosin (H&E) histopathological staining performed on a biopsy derived from a patient during the initial diagnosis of PDAC. The slide was scanned using a Leica DMI8 inverted fluorescence microscope. Morphologically abnormal ducts lined with cancerous cells (green arrows) are surrounded by a desmoplastic environment (pale purple staining).

The devastating pancreatic ductal adenocarcinoma (PDAC) 254,255 has a unique TME characterized by hyperactivated stromal fibroblasts, effective immunosuppression, and an elevated dense extracellular matrix (ECM) deposition known as desmoplasia 256 (Figure 4). This physical barrier, promoted by high levels of autophagy 257-259, limits the delivery and efficacy of chemotherapy 260 and immunotherapy 261, while autophagy supports immune evasion 101,102. Drug encapsulation has been tested to help penetrate this dense desmoplastic ECM barrier and target stromal cancer associated fibroblasts (CAFs) and tumor cells 262,263. PDAC cells overexpress integrins α_v_β_6_ 264 and α_v_β_3_ 234,265 and thus are good targets for RGD-decorated NPs. The CQ derivative hydroxychloroquine (HCQ, Figure 3) has similar lysosomotropic properties and anti-cancer modes of action to CQ by alkalinizing lysosomes and inhibiting autophagy 193,266. HCQ has been shown to be ~40% less toxic in animals 267 and have less adverse effects in humans 268, and has been clinically explored in the treatment of PDAC (Table S1) 269-274. For example, pre-operative treatment of PDAC patients with gemcitabine and HCQ markedly increased the overall survival in patients who had a > 51% increase in the autophagy marker LC3-II in circulating peripheral blood mononuclear cells (34.8 vs. 10.8 months, *p* < 0.05) 275. Moreover, HCQ was shown to possess antifibrotic activity, by reducing collagen levels and inhibiting ECM synthesis in 4T1 mouse tumor models 258. These findings led to the design of TR-PTX/HCQ-Lip, liposome-based NPs decorated with a multifunctional tandem peptide TH-RGD (TR), loaded with a combination of HCQ and PTX 276. TR consists of a targeting cyclic RGD tripeptide and a pH-responsive cell-penetrating peptide (CPP) 277. CPP should become protonated under the acidic pH of the TME, thus converting its charge from negative to positive and facilitating its membrane penetration by electrostatic forces, particularly between the positive charge of the CPP and the negatively charged polar head groups of membrane phospholipids 278,279. This enhances the RGD-based specificity of the liposomes to tumors. The ability of TR-PTX/HCQ-Lip to penetrate the dense fibrotic stroma and target the PDAC tumor was verified using a murine BxPC-3/NIH 3T3 heterogenous pancreatic tumor model 276. The heterogenous tumor model consisting of both CAFs and tumor cells is utilized to mimic the complex PDAC architecture and desmoplastic components 280. In comparison with free HCQ and non-targeted HCQ-containing NPs (PEG-HCQ-Lip, PEG-PTX/HCQ-Lip), TR-PTX/HCQ-Lip completely disrupted lysosomal accumulation of Lysotracker, and robustly inhibited autophagy in BxPC-3 and NIH 3T3 cells 276. Autophagy inhibition and reduction in ECM deposition were also verified *in vivo* in harvested tumors. Moreover, TR-PTX/HCQ-Lip was superior in inhibiting migration and invasion of BxPC-3 cells. In an orthotopic BxPC-3 tumor bearing mouse model, administration of free PTX+HCQ had little effect on tumor growth, eliciting a reduction of ~15% in tumor growth. In contrast, TR-PTX/HCQ-Lip exerted a dramatic growth inhibitory effect, resulting in an ~85% reduced tumor weight (*p* < 0.001). Consistent results were obtained with the heterogenous tumor mouse model, suggesting good response by CAFs. Moreover, TR-PTX/HCQ-Lip completely eliminated any surface liver metastases, compared to 50-90 metastatic nodules on livers of mice treated with free PTX+HCQ (*p* < 0.001) 276. In accord with the loss of body weight as a hallmark of PDAC 281, all orthotopic BxPC-3 tumor bearing mice exhibited weight loss with disease progression, while those treated with TR-PTX/HCQ-Lip largely retained their original weight. Importantly, the encapsulation of PTX and HCQ prevented hepatic toxicity induced by the free drugs 276. Collectively, these findings reveal a good response to HCQ as a drug adjuvant, and the advantages of drug encapsulation and tumor targeting.

Taking advantage of autophagy a step further, Yang Wang *et al.,* decided to not only prevent the beneficial impacts of autophagy in tumors, but to also use autophagy as a tumor killing approach. By both enhancing the first step and inhibiting the last stage of autophagy, the researchers induced autophagic catastrophic vacuolization and death of both tumor cells *in vitro* and mouse tumor models *in vivo* 282. This was achieved by utilizing a TAT-Beclin 1 peptide (T-B) along with HCQ-loaded liposomes (HCQ-Lip). The T-B peptide consists of the transduction domain of the CPP TAT protein, linked to the HIV-1 Nef-binding domain of Beclin 1 required to initiate autophagy 283. Indeed, inducing the generation of autophagosomes by T-B and preventing their fusion with lysosomes via HCQ-Lip, led to the overwhelming synergistic accumulation of autophagosomes (6-7-fold over free HCQ or T-B) in four different tumor cell lines. This led to 95% apoptosis/necrosis of cancer cells 282. Treatment of 4T1 xenograft-bearing BALB/C mice with an intra-tumoral injection of T-B and an intravenous injection of HCQ-Lip resulted in ~90% smaller tumors than in the control group (*p* < 0.001), which were 3.3-fold smaller than those of mice treated with each component alone (*p* < 0.001). Examination of the resected tumors revealed vast autophagic vacuolization and necrotic areas at the center of the tumors 282. Since HCQ is less toxic than CQ, all currently ongoing clinical trials, utilizing autophagy inhibitors to improve the outcome of cancer therapy, include HCQ (Table 1).

### Lys05

A CQ derivative that has gained much interest is Lys05 (Figure 3), a bisaminoquinoline dimeric form of CQ which is 10-fold more efficacious as an autophagy inhibitor than HCQ in human GBM LN229 cells 284. At high doses, Lys05 is such a potent autophagy inhibitor, that it elicited in mice an intestinal phenotype resembling genetic defects in the autophagy gene ATG16L1 284. Unlike HCQ, Lys05 was shown to have single-agent antitumor activity without untoward toxicity in mice bearing HT-29 CRC xenografts at low doses (i.e., 10 mg/kg and 40 mg/kg), hence achieving the goal of preventing adverse effects 284. The mode of action of Lys05 was demonstrated using the GBM U251 and LN229 cell lines 44. Following its accumulation in lysosomes, Lys05 induced LMP, resulting in lysosomal alkalinization and content release, resulting in mitochondrial depolarization and tumor cell death. While Lys05-induced lysosomal dysfunction did not prevent the fusion of lysosomes with autophagosomes, the degradation within autolysosomes was impaired, hence inhibiting the autophagy flux 44. In accord with the immune-suppressive effects of irradiation detailed above, irradiation of U251 and LN229 cells resulted in increased CTSB activity. In this respect, Lys05 was shown to robustly enhance the cytotoxic effect of irradiation *in vitro* via LMP and elevated irradiation-induced DNA damage 44.

### Overcoming resistance to tyrosine kinase inhibitors

Tyrosine kinase inhibitors (TKIs) have truly revolutionized the treatment of human malignancies 285-287. However, the efficacy of cancer treatment with TKIs has been hampered by the frequent emergence of multiple mechanisms of TKI resistance 288-290. In this respect, various TKIs are hydrophobic weak bases which highly accumulate in lysosomes, thereby being sequestered away from their kinase target 291,292. In fact, many TKIs were shown to enhance protective autophagy 293-299 which constitutes a major resistance mechanism 293,298.

Clear cell ovarian carcinoma (CCOC) is a subtype of ovarian cancer characterized by intrinsic chemoresistance, including to established TKIs 300,301. Sunitinib, a small-molecule multi-targeted TKI 302,303, initially elicited a good response in two CCOC patients 304, but had little effect in a clinical trial 305. A suggested mechanism of sunitinib resistance was its accumulation and sequestration in lysosomes 306-308, a mechanism that could be exploited for LCD by PDT 309,310. At low concentrations, sunitinib was shown to impair autophagy, however at clinically relevant cytotoxic drug levels, sunitinib increased the autophagy flux 294,295. Thus, several studies have shown the benefit of combining sunitinib treatment with an autophagy inhibitor 311-314, as was the case with CCOC 315. The combination of sunitinib and Lys05 exerted a synergistic growth inhibitory effect on three CCOC cell lines compared to each drug alone; an effect that was recapitulated by combining sunitinib with autophagy protein 5 (ATG5) siRNA. The effect of the combined treatment was further explored *in vivo* in heterotopic murine models bearing the human CCOC cells TOV21G and OVTOKO. Mice receiving this combination treatment exhibited a substantial reduction of 45% (*p* < 0.01) and 54% (*p* < 0.0001) in tumor growth compared with mice treated with monotherapy of sunitinib or Lys05, respectively 315.

Chronic myeloid leukemia (CML) is successfully treated with TKIs including imatinib, nilotinib, dasatinib, bosutinib and the newer TKI asciminib 316,317. However, leukemic stem cells (LSCs) are insensitive to TKIs and persist as a minimal residual disease (MRD) source, resulting in relapse 318-320. In this respect, inhibition of autophagy has been shown to sensitize CML cells to TKIs 321,322. Using a CML patient-derived xenograft model, Pablo Baquero *et al.,* showed that hematopoietic LSCs exhibit an increased autophagic flux compared to non-leukemic cells 323. Remarkably, treatment of these leukemic mice with Lys05 resulted in autophagy inhibition, while HCQ had no inhibitory effect. The latter results were recapitulated in stem cell-enriched (CD34^+^) cells isolated from CML patients. Lys05 induced autophagy inhibition, which reduced LSCs quiescence and promoted myeloid cell expansion and maturation in the CML mouse model 323. Lastly, the combination of Lys05 and nilotinib, a second generation TKI 324,325 that was shown to induce autophagy 296,297, resulted in a significant additive therapeutic effect by reducing the fraction of human CD45^+^ cells in the bone marrow of these CML mouse model (*p* = 0.05), while HCQ had no additive pharmacological effect 323. CD45 is a pan-leukocyte marker expressed on nearly all hematopoietic cells, including hematopoietic stem cells 326.

The most common type of leukemia, chronic lymphocytic leukemia (CLL), accounts for ~33% of newly diagnosed leukemias in the US 327,328. Survival of CLL cells relies on B-cell receptor (BCR) signaling 329,330, which is conveyed through various kinases, including the pivotal Bruton's tyrosine kinase (BTK) 331,332. While the TKI ibrutinib, an irreversible inhibitor of BTK 333,334 which induces autophagy 335,336, is considered to have revolutionized CLL treatment, patients still present acquired drug resistance and low complete remission rates 337,338. Various studies have demonstrated the hypersensitivity of CLL cells to LDs in comparison to healthy B-cells 339-341. Hence, the combination of ibrutinib with lysosome-sensitizing agents has been explored 342,343. This included the repurposing of widely used cationic amphiphilic antihistamines (CAAs) which have recently been recognized as LDs 60,63,69,343-347. These CAAs, including for example desloratadine, clemastine, and ebastine, bear a specific chemical structure containing hydrophobic rings and a hydrophilic amine group. This structure allows them on the one hand to traverse cell membranes and on the other hand undergo accumulation in acidic lysosomes upon amine group protonation.

## Sonodynamic therapy

RDT using X ray has an advantage of deep tissue penetration, enabling tumor targeting throughout the body 348. However, irradiation has inevitable side effects including secondary tumors induced by this mutagenic treatment 349, radiation-induced vasculopathy 350, cardiovascular disorders 351, and serious fatigue 352, all of which limit the biomedical application of RDT. PDT, which combines light energy with a wavelength compatible photosensitizer, is considered a well-established method for cancer treatment, including via lysosomal damage 71-76. However, PDT has many limitations and disadvantages 353,354; primarily, near-infrared-based PDT laser has poor tissue penetration (~1-5 mm) 355,356, requires photosensitizers at specific wavelengths and induces serious photosensitivity of healthy tissues like the skin 357.

In comparison to RDT and PDT, US-based sonodynamic therapy (SDT) has high tissue penetration capacity (> 10 cm) 358 and displays negligible side effects 359. SDT is useful for drug delivery 360,361, including for the temporary opening of the BBB 362 to facilitate delivery of drugs for the treatment of various CNS malignancies 363 including GBM 364 and brain metastases 365. SDT is also used with various sonosensitizers to generate cytotoxic ROS for cancer therapeutics 359,365,366, including immunotherapy 367-371, eliciting the desirable abscopal effect 366. However, as observed with other anti-cancer treatment modalities, SDT can induce autophagy which mitigates the anti-cancer therapeutic effects 372-375. Thus, combining SDT with lysosome-targeted autophagy inhibitors restores tumor sensitivity to treatment 376-379, and induces LMP-driven ICD, stimulating an adaptive immune response 114.

Achieving both inhibition of autophagy and LMP using SDT was demonstrated by Yong Liu *et al.* 379. This group implemented an innovative NP strategy by using a piezoelectric material, which can convert mechanical pressure into electrical energy 380,381, to generate Ba_0.85_Ca_0.15_Zr_0.1_Ti_0.9_O_3_ (BCZT) NPs 379. Following endocytosis, these BCZT NPs localized within lysosomes of murine B16 melanoma cells, where they had only minor deleterious effects on lysosomal function. However, during a 6 min application of 1.5 W/cm^2^ US, the US-mediated mechanical forces 382 caused the charge centers within the BCZT NPs to shift, resulting in a dipole moment 383 and consequent formation of (e-) electrons on their surface 379. These electrons interacted with the abundant protons (H^+^) in the lysosomes to produce hydrogen gas (H_2_), thereby drastically alkalinizing the lysosomes. This resulted in a 75% reduction in lysosomal acid phosphatase activity and robust LMP, leading to autophagy inhibition and rapid cell death. The combination of BCZT NPs + US was also tested *in vivo* on B16 tumor-bearing mice, where these NPs consistently accumulated in the tumor's lysosomes. The combined therapy with BCZT NPs + US displayed an efficacious 86.1% tumor suppression rate (*p* < 0.0001), while the BCZT NPs alone elicited a non-significant 10% reduction in tumor volume. This allows for US-directed targeted therapy, even if the NPs themselves are not tumor specific 379.

An additional piezo-sonosensitizer was designed by Xianbo Wu *et al.,* who synthesized a novel O_2_ self-sufficient Ir-C_3_N_5_ nanocomplex, composed of a nitrogen-rich carbon nitride (C_3_N_5_) nanosheet and 30% iridium(III) 384. Ir-C_3_N_5_ exhibited a strong dipole moment and consequent high piezoelectric catalytic performance under US. The surface electrons reacted with O_2_ to generate singlet oxygen (^1^O_2_), and the intermediate ·O_2_^-^ reacted with additional electrons to form H_2_O_2_, followed by H_2_O_2_ decomposition to generate ·OH, thus producing high ROS levels. Moreover, as a self-sufficient O_2_ producer, Ir-C_3_N_5_ can be used under hypoxic conditions, which exist in both solid and hematological malignancies 385. When incubated with human A-375 melanoma cells, Ir-C_3_N_5_ was shown to accumulate in lysosomes, though no explanation was given to this specific organelle targeting 384. However, since the accumulation in lysosomes was not time dependent, this probably did not occur via endocytosis, and the asymmetric structure of the C_3_N_5_ component with positive and negative charge centers, probably conferred lysosomotropic characteristics. Upon US activation (0.5W, 1 MHz, 3 min), lysosomal Ir-C_3_N_5_ generated high levels of ROS leading to LMP. The latter induced robust autophagy inhibition and cell death, ~70% apoptosis and necrosis for Ir-C_3_N_5_ + US vs ~10% for Ir-C_3_N_5_ and only ~3% in the control group. To verify that Ir-C_3_N_5_ + US induced ICD, the authors conducted an *in vitro* transwell macrophage polarization assay. As controls for M1 and M2 polarization, mouse J774A.1 macrophages were incubated with the canonical stimuli LPS and IL-4 for M1 and M2, respectively. Apoptotic A-375 cells following treatment with Ir-C_3_N_5_ + US, stimulated M1 polarization as strong as LPS, including the secretion of the pro-inflammatory cytokines TNFα and IL-6. Using subcutaneous murine B16-F10 tumor models, Ir-C_3_N_5_ + US was shown to stimulate CTLs infiltration into the tumor and lymph nodes. This stimulation resulted in the complete inhibition of both the primary tumor and distant tumor growth as well as eliminated lung metastases, suggesting an efficient abscopal effect. The combined treatment with Ir-C_3_N_5_ + US increased the survival rate of the mice beyond the scope of the experiment (> 50 days) 384.

## Future perspectives

The current review highlights the burning necessity of simultaneously targeting tumor cells as well as the TME and tumor immune microenvironment (TIME) 386. Targeting only tumor cells often results in chemoresistance, as the TME and TIME actively promote tumor cell survival, growth, invasion, immune evasion and metastasis 387-391. Lysosomal modulating agents that impair autophagy simultaneously target the tumor and its TME and TIME, while minimizing adverse effects. Targeting lysosomes of TAMs and stroma cells can convert an immunosuppressive microenvironment into an immune-supportive one, enhancing the efficacy of immunotherapies. Several clinical trials using CQ/HCQ in combination therapy, have shown great promise (Table S1) 53 and warrant further dedicated studies. This is particularly relevant in high mortality cancer types with no efficacious therapy like PDAC. As abovementioned, PDAC is characterized by a highly dense desmoplasia. The latter is present in other types of tumors, e.g., cervical cancer 392, breast cancer 393, lung cancer 394, squamous cell carcinoma 395 and small intestine neuroendocrine tumors 396, leading to chemoresistance and dismal prognosis 392,395,397-399. Thus, it is paramount to overcome this desmoplastic barrier for efficient cancer eradication. In this regard, recent studies reveal that desmoplasia is promoted by autophagy 257-259. Hence, we find autophagy inhibition via lysosomal targeting a promising therapeutic strategy. Figure 4 illustrates the role of autophagy in shaping the TME and TIME of PDAC, and the reversal effect of autophagy inhibition by lysosome targeting agents. The anti-fibrotic activity of HCQ inhibits ECM synthesis by reducing the secretion of collagen and hyaluronan by stromal fibroblasts 258,276. The polarization of macrophages to the M1 phenotype reduces the levels of TGF-β which promotes autophagy and ECM stiffness 400. Reduction in desmoplasia along with immune stimulation restores chemotherapeutic drug accessibility to the tumor and chemosensitivity, as well as an enhanced anti-tumor immune response. It should be noted that HCQ is used in this demonstration since it was shown to reduce desmoplasia in murine models 258,276. However, since an HCQ dose of 1,200 mg/day is required to induce inhibition of autophagy in cancer patients 275,401, a dose that can elicit grade 3-4 adverse effects 401-403, other more potent lysosome disrupting agents should be tested, or HCQ should be encapsulated. A potential candidate as an HCQ substitute is the FDA approved TKI nintedanib 404. From a mechanistic perspective, nintedanib blocks multiple tyrosine kinase receptors including VEGFR, PDGFR, and FGFR, which are paramount in the signaling pathways which culminate in pathological lung fibrosis. Nintedanib accumulates in the membrane of lysosomes and disrupts their integrity and function 59,405,406, leading to autophagy inhibition and autophagic cell death 405,407. Consistently, nintedanib has displayed potent antifibrotic activities that reprogram the TME by reducing ECM secretion by CAFs as well as enhancing immunity and reducing TGF-β levels 408-410. While these abilities are exploited for the treatment of lung fibrosis 411,412, nintedanib is still primarily referred to as a TKI and not as a *bona fide* lysosomotropic agent. Based on the established anti-fibrotic activity of nintedanib, clinical trials are warranted that will explore its plausible antitumor activity as a desmoplasia inhibitor, as monotherapy or in combination with other chemotherapeutics, in desmoplastic cancers like PDAC. In this respect, a clinical trial has been already conducted in PDAC (NCT02902484).

**Figure 5:**
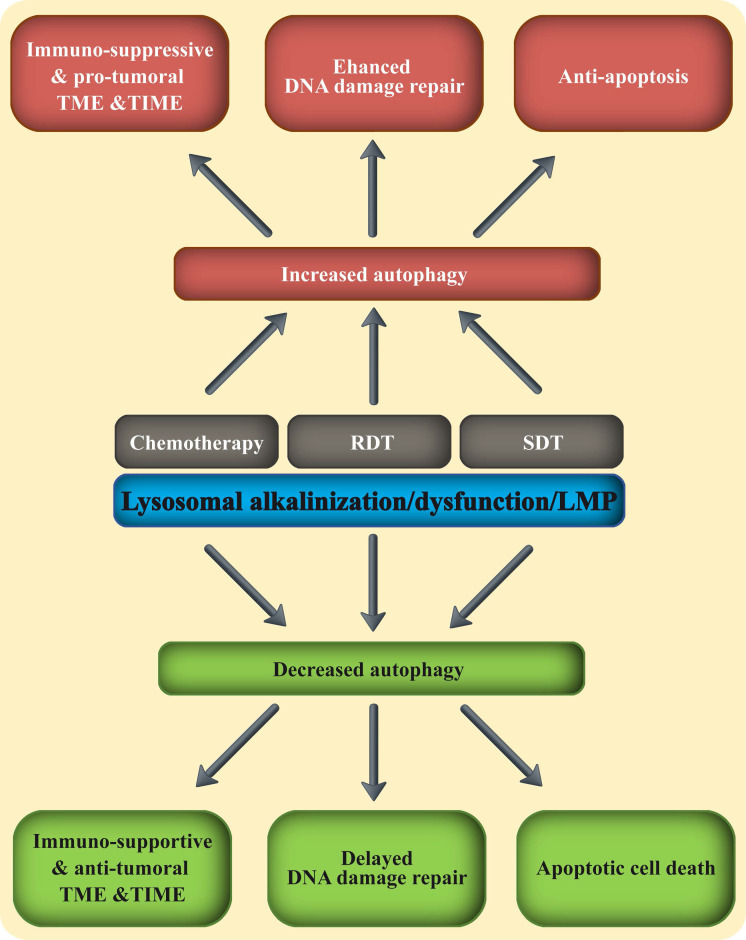
** Combining lysosomal disrupting agents with the three fundamental anti-cancer treatment modalities.** The three main anti-cancer therapeutic modalities including chemotherapy, RDT and SDT, were all shown to increase autophagy in malignant and immune cells. This enhanced autophagy mitigates their therapeutic activity, by promoting an immune-suppressive TME & TIME and immune evasion, upregulated DNA damage response and repair, as well as anti-apoptosis. Adding a lysosomal disrupting agent to any of these three therapeutic modalities, blocks autophagy and reverses the pro-tumoral TME & TIME to anti-tumor environment.

In a recent review, Stephanie Nagy *et al.,* retrospectively analyzed the impact of CAAs on the efficiency of immune checkpoint inhibitors (ICIs)-based immunotherapy in cancer patients 413. The six studies included in this analysis, encompassing 4,171 patients with different types of malignancies, showed great potential. Collectively, patients who received a combination of CAAs and ICIs displayed a significant improvement in overall survival rates and longer progression-free survival rates compared to patients who did not receive antihistamines 413. Considering the minimal adverse effects of second generation CAAs 414, and their ability to induce lysosomal alkalinization and LMP 69,343,345,347, further clinical evaluation should be performed. The same can be conveyed about ginsenosides which exhibit multiple beneficial properties, including health improvement, anti-tumor activity and enhanced immunity. Thus, drug repurposing to generate efficacious combination therapies targeting the tumor as well as the stroma should be in the epicenter of future innovative therapeutic drug development efforts (Figure 5).

## Supplementary Material

Supplementary table.

## Figures and Tables

**Table 1:** ** Ongoing clinical trials using a lysosome disrupting agent as an autophagy inhibitor to enhance cancer treatment outcome.** All clinical trials utilized hydroxychloroquine in addition to the base therapy.

Clinical trial	Cancer type	Base therapy mode	ClinicalTrials.gov identifier
Phase I/II	Solid tumors	Combinations of metformin, sirolimus, dasatanib, nelfinavir	NCT05036226
Phase II	Refractory solid tumors	Devimistat and 5-Fluorouracil or gemcitabine	NCT05733000
Phase I	Advanced solid tumors	MK2206 (highly selective AKT inhibitor)	NCT01480154
Proof of principle	Prostate cancer	A placebo-controlled study	NCT06408298
Phase II	Serous ovarian cancer	Nelfinavir mesylate, bevacizumab	NCT06971744
Interventional	Nasopharyngeal carcinoma	Chemotherapy and radiotherapy pretreatment	NCT06389201
Phase I/II	Recurrent brain tumors	Dabrafenib and/or trametinib	NCT04201457
Phase I/II	PDAC	mFOLFIRINOX	NCT04911816
Phase 0/I	PDAC	Paricalcitol and losartan	NCT05365893
Phase II	Metastatic PDAC	Trametinib	NCT05518110
Phase I	Metastatic PDAC	Trametinib	NCT03825289
Phase I	Metastatic PDAC	Chlorphenesin Carbamate and mFOLFIRINOX	NCT05083780
Phase II	Metastatic PDAC	Paricalcitol, gemcitabine and nab-paclitaxel	NCT04524702
Phase II	Advanced NSCLC	Erlotinib	NCT00977470
Phase II	Metastatic CRC	5-Fluorouracil, irinotecan and bevacizumab	NCT05843188
Phase II	Metastatic CRC	Encorafenib and cetuximab or panitumumab	NCT05576896
Phase I/II	Recurrent osteosarcoma	Gemcitabine and docetaxel	NCT03598595
Phase I/II	Metastatic melanoma	Nivolumab or nivolumab/​ipilimumab	NCT04464759
Phase I/II	Advanced breast cancer	Trastuzumab deruxtecan or sacituzumab govitecan	NCT06328387
Phase II	Dormant breast cancer	Palbociclib	NCT04841148
Phase II	Dormant breast cancer	Everolimus	NCT03032406

Abbreviations: CRC: colorectal cancer. mFOLFIRINOX: modified FOLFIRINOX using 80% of drug doses; NSCLC: non-small cell lung cancer; PDAC: pancreatic ductal adenocarcinoma.

## Abbreviations

5-FU: 5-Fluorouracil
APCs: antigen presenting cells
aRCT: adjuvant-radiochemotherapy
ATG5: autophagy protein 5
Au@Cu2-xSe: core-shell copper selenide-coated gold NPs
BafA1: bafilomycin A1
BBB: blood-brain-barrier
BCR: B-cell receptor
BCZT: Ba_0.85_Ca_0.15_Zr_0.1_Ti_0.9_O_3_
BEV: bevacizumab
bLDH: MgAl-based hydrotalcite
BMDMs: bone marrow derived macrophages
BTK: Bruton's tyrosine kinase
CAFs: cancer associated fibroblasts
CAAs: cationic amphiphilic antihistamines
CCOC: clear cell ovarian carcinoma
CHEK1: checkpoint kinase 1
CLL: chronic lymphocytic leukemia
CML: chronic myeloid leukemia
CpG: cytidine monophosphate guanosine oligodeoxynucleotide
CPP: cell-penetrating peptide
CQ: chloroquine
CRC: colorectal cancer
CTLs: cytotoxic T-lymphocytes, CD8^+^ T cells
CTSB: cathepsin B
DDR: DNA damage repair
DHA: dihydroartemisinin
ECM: extracellular matrix
ER: endoplasmic reticulum
ESR2: estrogen receptor 2
GBM: glioblastoma multiforme
GICs: glioma initiating cells
GLUT1: glucose transporter 1
H&E: hematoxylin and eosin
HCQ: hydroxychloroquine
HCQ-Lip: HCQ-loaded liposomes
ICD: immunogenic cell death
ICIs: immune checkpoint inhibitors
IFN-γ: interferon gamma
iNOS: inducible nitric oxide synthase
IQ: imidazoquinoline
Ir-C3N5: nitrogen-rich carbon nitride nanosheet and iridium(III)
LCD: lysosomal cell death
LDs: lysosomotropic drugs
LMP: lysosomal membrane permeabilization
LNPs: lipid NPs
LPS: lipopolysaccharide
LSCs: leukemic stem cells
LTA: lipoteichoic acid
Mcoln1: mucolipin
MDSCs: myeloid-derived suppressor cells
MRD: minimal residual disease
MSNs: mesoporous silica NPs
NCF1: neutrophil cytosolic factor 1
NK: natural killer
NP: nanoparticle
NSCLC: non-small cell lung cancer
PDA: polydopamine
PDAC: pancreatic ductal adenocarcinoma
PDGF: platelet-derived growth factor
PDT: photodynamic therapy
PFS: progression free survival
PGN: pH-gated nanoadjuvant
PGNs: pH-gated nanoparticles
PTX: paclitaxel
RDT: radiation therapy, radiotherapy
R_g3_-LPs: ginsenoside R_g3_ liposomes
rGBM: recurrent GBM
RGD: arginyl-glycyl-aspartic acid tripeptide
R_h2_-lipo: ginsenosides R_h2_ liposome
RLNP/DC: LNPs co-loaded with CQ and DHA, and decorated with RGD
SDT: sonodynamic therapy
TAMs: tumor associated macrophages
T-B: TAT-Beclin 1 peptide
TDLN: tumor-draining lymph nodes
TFP: trifluoperazine
TGF-β: transforming growth factor β
TIME: tumor immune microenvironment
TKIs: tyrosine kinase inhibitors
TLR: toll like receptor
TME: tumor microenvironment
TMZ: temozolomide
TNBC: triple negative breast cancer
TNFα: tumor necrosis factor α
TR: a targeting cyclic RGD tripeptide with a pH-responsive CPP
Tregs: regulatory T cells
TR-PTX/HCQ-Lip: liposome-based NPs decorated with TR, loaded with HCQ and PTX
UIO: ultrasmall iron oxide
UIOQM-IQ: UIO NP conjugated to a CTSB-cleavable peptide, an aggregation-induced emission fluorophore, and surface IQ
US: ultrasound
VEGF: vascular endothelial growth factor
ROS: reactive oxygen species

## References

[1] Ohkuma S, Poole B (1978). Fluorescence probe measurement of the intralysosomal pH in living cells and the perturbation of pH by various agents. Proc Natl Acad Sci U S A.

[2] Johnson DE, Ostrowski P, Jaumouillé V, Grinstein S (2016). The position of lysosomes within the cell determines their luminal pH. J Cell Biol.

[3] Webb BA, Aloisio FM, Charafeddine RA, Cook J, Wittmann T, Barber DL (2021). pHLARE: A new biosensor reveals decreased lysosome pH in cancer cells. Mol Biol Cell.

[4] Hu Y, Wang X, Lu K, Cheang C, Liu Y, Zhu Y (2026). Aggregation-induced emission of DNA fluorescence as a novel pan-marker of cell death, senescence and sepsis *in vitro* and *in vivo*. Theranostics.

[5] Schröder BA, Wrocklage C, Hasilik A, Saftig P (2010). The proteome of lysosomes. Proteomics.

[6] Ratto E, Chowdhury SR, Siefert NS, Schneider M, Wittmann M, Helm D (2022). Direct control of lysosomal catabolic activity by mTORC1 through regulation of V-ATPase assembly. Nat Commun.

[7] Yoon MC, Hook V, O'Donoghue AJ (2022). Cathepsin B Dipeptidyl Carboxypeptidase and Endopeptidase Activities Demonstrated across a Broad pH Range. Biochemistry.

[8] Abe A, Shayman JA (1998). Purification and characterization of 1-O-acylceramide synthase, a novel phospholipase A2 with transacylase activity. J Biol Chem.

[9] Patra S, Patil S, Klionsky DJ, Bhutia SK (2023). Lysosome signaling in cell survival and programmed cell death for cellular homeostasis. J Cell Physiol.

[10] Ballabio A, Bonifacino JS (2020). Lysosomes as dynamic regulators of cell and organismal homeostasis. Nat Rev Mol Cell Biol.

[11] Settembre C, Perera RM (2024). Lysosomes as coordinators of cellular catabolism, metabolic signalling and organ physiology. Nat Rev Mol Cell Biol.

[12] Zhang Z, Yue P, Lu T, Wang Y, Wei Y, Wei X (2021). Role of lysosomes in physiological activities, diseases, and therapy. J Hematol Oncol.

[13] Inpanathan S, Botelho RJ (2019). The Lysosome Signaling Platform: Adapting With the Times. Front cell Dev Biol.

[14] Lawrence RE, Zoncu R (2019). The lysosome as a cellular centre for signalling, metabolism and quality control. Vol. 21, Nature Cell Biology. Nature Publishing Group.

[15] Eriksson I, Öllinger K (2024). Lysosomes in Cancer—At the Crossroad of Good and Evil. Cells.

[16] Davidson SM, Vander Heiden MG (2017). Critical Functions of the Lysosome in Cancer Biology. Annu Rev Pharmacol Toxicol.

[17] Mahapatra KK, Mishra SR, Behera BP, Patil S, Gewirtz DA, Bhutia SK (2021). The lysosome as an imperative regulator of autophagy and cell death. Cell Mol Life Sci.

[18] Nanayakkara R, Gurung R, Rodgers SJ, Eramo MJ, Ramm G, Mitchell CA (2023). Autophagic lysosome reformation in health and disease. Autophagy.

[19] Kuchitsu Y, Taguchi T (2024). Lysosomal microautophagy: an emerging dimension in mammalian autophagy. Trends Cell Biol.

[20] Trelford CB, Di Guglielmo GM (2021). Molecular mechanisms of mammalian autophagy. Biochem J.

[21] Vargas JNS, Hamasaki M, Kawabata T, Youle RJ, Yoshimori T (2023). The mechanisms and roles of selective autophagy in mammals. Nat Rev Mol Cell Biol.

[22] Chen T, Tu S, Ding L, Jin M, Chen H, Zhou H (2023). The role of autophagy in viral infections. J Biomed Sci.

[23] Yan X, Zhou R, Ma Z (2019). Autophagy—Cell Survival and Death. Adv Exp Med Biol.

[24] Bata N, Cosford NDP (2021). Cell Survival and Cell Death at the Intersection of Autophagy and Apoptosis: Implications for Current and Future Cancer Therapeutics. ACS Pharmacol Transl Sci.

[25] Das S, Shukla N, Singh SS, Kushwaha S, Shrivastava R (2021). Mechanism of interaction between autophagy and apoptosis in cancer. Apoptosis.

[26] Yu G, Klionsky DJ (2022). Life and Death Decisions—The Many Faces of Autophagy in Cell Survival and Cell Death. Biomolecules.

[27] Liu SZ, Yao SJ, Yang H, Liu SJ, Wang YJ (2023). Autophagy: Regulator of cell death. Cell Death Dis.

[28] He C (2022). Balancing nutrient and energy demand and supply via autophagy. Curr Biol.

[29] Feng Y, Chen Y, Wu X, Chen J, Zhou Q, Liu B (2024). Interplay of energy metabolism and autophagy. Autophagy.

[30] Lemasters JJ (2014). Variants of mitochondrial autophagy: Types 1 and 2 mitophagy and micromitophagy (Type 3). Redox Biol.

[31] Fu X, Cui J, Meng X, Jiang P, Zheng Q, Zhao W (2021). Endoplasmic reticulum stress, cell death and tumor: Association between endoplasmic reticulum stress and the apoptosis pathway in tumors (Review). Oncol Rep.

[32] Merighi A, Lossi L (2022). Endoplasmic Reticulum Stress Signaling and Neuronal Cell Death. Int J Mol Sci.

[33] Chipurupalli S, Samavedam U, Robinson N (2021). Crosstalk Between ER Stress, Autophagy and Inflammation. Front Med.

[34] Qin Y, Ashrafizadeh M, Mongiardini V, Grimaldi B, Crea F, Rietdorf K (2023). Autophagy and cancer drug resistance in dialogue: Pre-clinical and clinical evidence. Cancer Lett.

[35] Niu X, You Q, Hou K, Tian Y, Wei P, Zhu Y (2025). Autophagy in cancer development, immune evasion, and drug resistance. Drug Resist Updat.

[36] Hu X, Wen L, Li X, Zhu C (2022). Relationship Between Autophagy and Drug Resistance in Tumors. Mini-Reviews Med Chem.

[37] Qiang L, Zhao B, Shah P, Sample A, Yang S, He YY (2016). Autophagy positively regulates DNA damage recognition by nucleotide excision repair. Autophagy.

[38] Eliopoulos AG, Havaki S, Gorgoulis VG (2016). DNA Damage Response and Autophagy: A Meaningful Partnership. Front Genet.

[39] Ambrosio S, Majello B (2020). Autophagy Roles in Genome Maintenance. Cancers (Basel).

[40] Park C, Suh Y, Cuervo AM (2015). Regulated degradation of Chk1 by chaperone-mediated autophagy in response to DNA damage. Nat Commun.

[41] Elliott IA, Dann AM, Xu S, Kim SS, Abt ER, Kim W (2019). Lysosome inhibition sensitizes pancreatic cancer to replication stress by aspartate depletion. Proc Natl Acad Sci U S A.

[42] Zheng K, Li Y, Wang S, Wang X, Liao C, Hu X (2016). Inhibition of autophagosome-lysosome fusion by ginsenoside Ro via the ESR2-NCF1-ROS pathway sensitizes esophageal cancer cells to 5-fluorouracil-induced cell death via the CHEK1-mediated DNA damage checkpoint. Autophagy.

[43] Saleh T, As Sobeai HM, Alhoshani A, Alhazzani K, Almutairi MM, Alotaibi M (2022). Effect of Autophagy Inhibitors on Radiosensitivity in DNA Repair-Proficient and -Deficient Glioma Cells. Medicina (Kaunas).

[44] Zhou W, Guo Y, Zhang X, Jiang Z (2020). Lys05 induces lysosomal membrane permeabilization and increases radiosensitivity in glioblastoma. J Cell Biochem.

[45] Debnath J, Gammoh N, Ryan KM (2023). Autophagy and autophagy-related pathways in cancer. Nat Rev Mol Cell Biol.

[46] Vitto VAM, Bianchin S, Zolondick AA, Pellielo G, Rimessi A, Chianese D (2022). Molecular Mechanisms of Autophagy in Cancer Development, Progression, and Therapy. Biomedicines.

[47] Turek K, Jarocki M, Kulbacka J, Saczko J (2021). Dualistic role of autophagy in cancer progression. Adv Clin Exp Med.

[48] Bhol CS, Senapati PK, Kar RK, Chew G, Mahapatra KK, Lee EHC (2025). Autophagy paradox: Genetic and epigenetic control of autophagy in cancer progression. Cancer Lett.

[49] Chen JL, Wu X, Yin D, Jia XH, Chen X, Gu ZY (2023). Autophagy inhibitors for cancer therapy: Small molecules and nanomedicines. Pharmacol Ther.

[50] Jain V, Singh MP, Amaravadi RK (2023). Recent advances in targeting autophagy in cancer. Trends Pharmacol Sci.

[51] Hama Y, Ogasawara Y, Noda NN (2023). Autophagy and cancer: Basic mechanisms and inhibitor development. Cancer Sci.

[52] Tonkin-Reeves A, Giuliani CM, Price JT (2023). Inhibition of autophagy; an opportunity for the treatment of cancer resistance. Front Cell Dev Biol.

[53] Mohsen S, Sobash PT, Algwaiz GF, Nasef N, Al-Zeidaneen SA, Karim NA (2022). Autophagy Agents in Clinical Trials for Cancer Therapy: A Brief Review. Curr Oncol.

[54] Klionsky DJ, Abdalla FC, Abeliovich H, Abraham RT, Acevedo-Arozena A, Adeli K (2012). Guidelines for the use and interpretation of assays for monitoring autophagy. Autophagy.

[55] Kimura S, Noda T, Yoshimori T (2007). Dissection of the autophagosome maturation process by a novel reporter protein, tandem fluorescent-tagged LC3. Autophagy.

[56] Pisonero-Vaquero S, Medina DL (2017). Lysosomotropic Drugs: Pharmacological Tools to Study Lysosomal Function. Curr Drug Metab.

[57] Mlejnek P, Havlasek J, Pastvova N, Dolezel P, Dostalova K (2022). Lysosomal sequestration of weak base drugs, lysosomal biogenesis, and cell cycle alteration. Biomed Pharmacother.

[58] Ellegaard AM, Bach P, Jäättelä M (2023). Targeting Cancer Lysosomes with Good Old Cationic Amphiphilic Drugs. Rev Physiol Biochem Pharmacol.

[59] Stark M, Silva TFD, Levin G, Machuqueiro M, Assaraf YG (2020). The Lysosomotropic Activity of Hydrophobic Weak Base Drugs is Mediated via Their Intercalation into the Lysosomal Membrane. Cells.

[60] Scrima S, Lambrughi M, Favaro L, Maeda K, Jäättelä M, Papaleo E (2024). Acidic sphingomyelinase interactions with lysosomal membranes and cation amphiphilic drugs: A molecular dynamics investigation. Comput Struct Biotechnol J.

[61] Paloncýová M, DeVane R, Murch B, Berka K, Otyepka M (2014). Amphiphilic drug-like molecules accumulate in a membrane below the head group region. J Phys Chem B.

[62] Fu D, Zhou J, Zhu WS, Manley PW, Wang YK, Hood T (2014). Imaging the intracellular distribution of tyrosine kinase inhibitors in living cells with quantitative hyperspectral stimulated Raman scattering. Nat Chem.

[63] Bagdonaitė R, Žvirblis R, Dodonova-Vaitkunienė J, Polita A (2025). Cancer Cell Identification via Lysosomal Membrane Microviscosities Using a Green-Emitting BODIPY Molecular Rotor. JACS Au.

[64] Seu KJ, Cambrea LR, Everly RM, Hovis JS (2006). Influence of lipid chemistry on membrane fluidity: tail and headgroup interactions. Biophys J.

[65] Zhitomirsky B, Yunaev A, Kreiserman R, Kaplan A, Stark M, Assaraf YG (2018). Lysosomotropic drugs activate TFEB via lysosomal membrane fluidization and consequent inhibition of mTORC1 activity. Cell Death Dis.

[66] Matyszewska D (2016). Comparison of the interactions of daunorubicin in a free form and attached to single-walled carbon nanotubes with model lipid membranes. Beilstein J Nanotechnol.

[67] Kornhuber J, Henkel AW, Groemer TW, Städtler S, Welzel O, Tripal P (2010). Lipophilic cationic drugs increase the permeability of lysosomal membranes in a cell culture system. J Cell Physiol.

[68] Ponsford AH, Ryan TA, Raimondi A, Cocucci E, Wycislo SA, Fröhlich F (2021). Live imaging of intra-lysosome pH in cell lines and primary neuronal culture using a novel genetically encoded biosensor. Autophagy.

[69] Liu B, Chen R, Zhang Y, Huang J, Luo Y, Rosthøj S (2023). Cationic amphiphilic antihistamines inhibit STAT3 via Ca2+-dependent lysosomal H+ efflux. Cell Rep.

[70] Qiu C, Xia F, Zhang J, Shi Q, Meng Y, Wang C (2023). Advanced Strategies for Overcoming Endosomal/Lysosomal Barrier in Nanodrug Delivery. Research.

[71] Adar Y, Stark M, Bram EE, Nowak-Sliwinska P, van den Bergh H, Szewczyk G (2012). Imidazoacridinone-dependent lysosomal photodestruction: a pharmacological Trojan horse approach to eradicate multidrug-resistant cancers. Cell Death Dis.

[72] Bacellar IOL, Tsubone TM, Pavani C, Baptista MS (2015). Photodynamic efficiency: From molecular photochemistry to cell death. Int J Mol Sci.

[73] Attar GS, Kumar M, Bhalla V (2024). Targeting sub-cellular organelles for boosting precision photodynamic therapy. Chem Commun.

[74] Calori IR, Bi H, Tedesco AC (2021). Expanding the Limits of Photodynamic Therapy: The Design of Organelles and Hypoxia-Targeting Nanomaterials for Enhanced Photokilling of Cancer. ACS Appl Bio Mater.

[75] Alzeibak R, Mishchenko TA, Shilyagina NY, Balalaeva I V, Vedunova M V, Krysko D V (2021). Targeting immunogenic cancer cell death by photodynamic therapy: past, present and future. J Immunother Cancer.

[76] Sharma VK, Stark M, Fridman N, Assaraf YG, Gross Z (2022). Doubly Stimulated Corrole for Organelle-Selective Antitumor Cytotoxicity. J Med Chem.

[77] Zhao W, Wang L, Zhang M, Liu Z, Wu C, Pan X (2024). Photodynamic therapy for cancer: mechanisms, photosensitizers, nanocarriers, and clinical studies. MedComm.

[78] Wang X, Peng J, Meng C, Feng F (2024). Recent advances for enhanced photodynamic therapy: from new mechanisms to innovative strategies. Chem Sci.

[79] Thorsson V, Gibbs DL, Brown SD, Wolf D, Bortone DS, Ou Yang TH (2018). The Immune Landscape of Cancer. Immunity.

[80] Wang Z, Katsaros D, Wang J, Biglio N, Hernandez BY, Fei P (2023). Machine learning-based cluster analysis of immune cell subtypes and breast cancer survival. Sci Rep.

[81] Reis-Sobreiro M, Teixeira da Mota A, Jardim C, Serre K (2021). Bringing macrophages to the frontline against cancer: Current immunotherapies targeting macrophages. Cells.

[82] Liu J, Geng X, Hou J, Wu G (2021). New insights into M1/M2 macrophages: key modulators in cancer progression. Cancer Cell Int.

[83] Boutilier AJ, Elsawa SF (2021). Macrophage polarization states in the tumor microenvironment. Int J Mol Sci.

[84] Saeed AF (2025). Tumor-Associated Macrophages: Polarization, Immunoregulation, and Immunotherapy. Cells.

[85] Chen S, Saeed AFUH, Liu Q, Jiang Q, Xu H, Xiao GG (2023). Macrophages in immunoregulation and therapeutics. Signal Transduct Target Ther.

[86] Müller E, Christopoulos PF, Halder S, Lunde A, Beraki K, Speth M (2017). Toll-Like Receptor Ligands and Interferon-γ Synergize for Induction of Antitumor M1 Macrophages. Front Immunol.

[87] Jung M, Bonavida B (2024). Immune Evasion in Cancer Is Regulated by Tumor-Asociated Macrophages (TAMs): Targeting TAMs. Crit Rev Oncog.

[88] Jackute J, Zemaitis M, Pranys D, Sitkauskiene B, Miliauskas S, Vaitkiene S (2018). Distribution of M1 and M2 macrophages in tumor islets and stroma in relation to prognosis of non-small cell lung cancer. BMC Immunol.

[89] Tiainen S, Tumelius R, Rilla K, Hämäläinen K, Tammi M, Tammi R (2015). High numbers of macrophages, especially M2-like (CD163-positive), correlate with hyaluronan accumulation and poor outcome in breast cancer. Histopathology.

[90] Hu H, Hang JJ, Han T, Zhuo M, Jiao F, Wang LW (2016). The M2 phenotype of tumor-associated macrophages in the stroma confers a poor prognosis in pancreatic cancer. Tumor Biol.

[91] Hadimani SM, Das S, Harish KG (2023). An immunohistochemical evaluation of tumor-associated macrophages (M1 and M2) in carcinoma prostate - An institutional study. J Cancer Res Ther.

[92] Chen D, Xie J, Fiskesund R, Dong W, Liang X, Lv J (2018). Chloroquine modulates antitumor immune response by resetting tumor-associated macrophages toward M1 phenotype. Nat Commun.

[93] Tang M, Chen B, Xia H, Pan M, Zhao R, Zhou J (2023). pH-gated nanoparticles selectively regulate lysosomal function of tumour-associated macrophages for cancer immunotherapy. Nat Commun.

[94] Ma J, Ma R, Zeng X, Zhang L, Liu J, Zhang W (2023). Lysosome blockade induces divergent metabolic programs in macrophages and tumours for cancer immunotherapy. J Exp Clin Cancer Res.

[95] Cui C, Chakraborty K, Tang XA, Schoenfelt KQ, Hoffman A, Blank A (2021). A lysosome-targeted DNA nanodevice selectively targets macrophages to attenuate tumours. Nat Nanotechnol.

[96] Huang SCC, Everts B, Ivanova Y, O'Sullivan D, Nascimento M, Smith AM (2014). Cell-intrinsic lysosomal lipolysis is essential for alternative activation of macrophages. Nat Immunol.

[97] Delamarre L, Pack M, Chang H, Mellman I, Trombetta ES (2005). Differential lysosomal proteolysis in antigen-presenting cells determines antigen fate. Science (80- ).

[98] Gao J, Ochyl LJ, Yang E, Moon JJ (2017). Cationic liposomes promote antigen cross-presentation in dendritic cells by alkalizing the lysosomal pH and limiting the degradation of antigens. Int J Nanomedicine.

[99] Wen JH, Li DY, Liang S, Yang C, Tang JX, Liu HF (2022). Macrophage autophagy in macrophage polarization, chronic inflammation and organ fibrosis. Front Immunol.

[100] Chen M, Wang J, Zhang P, Jiang Z, Chen S, Liang S (2025). Low molecular weight fucoidan induces M2 macrophage polarization to attenuate inflammation through activation of the AMPK/mTOR autophagy pathway. Eur J Pharmacol.

[101] Yamamoto K, Venida A, Yano J, Biancur DE, Kakiuchi M, Gupta S (2020). Autophagy promotes immune evasion of pancreatic cancer by degrading MHC-I. Nature.

[102] Xing X, Li XQ, Yin SQ, Ma HT, Xiao SY, Tulamaiti A (2025). OASL promotes immune evasion in pancreatic ductal adenocarcinoma by enhancing autolysosome-mediated degradation of MHC-I. Theranostics.

[103] Deng G, Zhou L, Wang B, Sun X, Zhang Q, Chen H (2022). Targeting cathepsin B by cycloastragenol enhances antitumor immunity of CD8 T cells via inhibiting MHC-I degradation. J Immunother Cancer.

[104] Allen SL, Lundberg AS (2011). Amonafide: A potential role in treating acute myeloid leukemia. Expert Opin Investig Drugs.

[105] Lehenkari PP, Kellinsalmi M, Näpänkangas JP, Ylitalo K V, Mönkkönen J, Rogers MJ (2002). Further insight into mechanism of action of clodronate: Inhibition of mitochondrial ADP/ATP translocase by a nonhydrolyzable, adenine-containing metabolite. Mol Pharmacol.

[106] Ruan J, Zheng H, Rong X, Rong X, Zhang J, Fang W (2016). Over-expression of cathepsin B in hepatocellular carcinomas predicts poor prognosis of HCC patients. Mol Cancer.

[107] Ma K, Chen X, Liu W, Chen S, Yang C, Yang J (2022). CTSB is a negative prognostic biomarker and therapeutic target associated with immune cells infiltration and immunosuppression in gliomas. Sci Rep.

[108] Fujimoto T, Tsunedomi R, Matsukuma S, Yoshimura K, Oga A, Fujiwara N (2021). Cathepsin B is highly expressed in pancreatic cancer stem-like cells and is associated with patients' surgical outcomes. Oncol Lett.

[109] Yan Z, Zheng G, Zou J, Zou X, Chai K, Zhang G (2025). Cathepsin B in urological tumors: unraveling its role and therapeutic potential. Discov Oncol.

[110] Shi Q, Shen Q, Liu Y, Shi Y, Huang W, Wang X (2022). Increased glucose metabolism in TAMs fuels O-GlcNAcylation of lysosomal Cathepsin B to promote cancer metastasis and chemoresistance. Cancer Cell.

[111] Chen Y, Chen Q, Ma Y, Su X, Zhang C, Li K Cathepsin B-Ignited Nanorocket To Blast Tumor Lysosomes for TLR-Fortified Lysosomal Immunotherapy with Dual-Switchable Fluorescence/Magnetic Resonance Imaging. J Am Chem Soc. 2025.

[112] Wang F, Gómez-Sintes R, Boya P (2018). Lysosomal membrane permeabilization and cell death. Traffic.

[113] Radulovic M, Yang C, Stenmark H Lysosomal membrane homeostasis and its importance in physiology and disease. Nat Rev Mol Cell Biol. 2025.

[114] Iulianna T, Kuldeep N, Eric F (2022). The Achilles' heel of cancer: targeting tumors via lysosome-induced immunogenic cell death. Cell Death Dis.

[115] Arimoto KI, Miyauchi S, Liu M, Zhang DE (2024). Emerging role of immunogenic cell death in cancer immunotherapy. Front Immunol.

[116] Ben-Nun Y, Merquiol E, Brandis A, Turk B, Scherz A, Blum G (2015). Photodynamic Quenched Cathepsin Activity Based Probes for Cancer Detection and Macrophage Targeted Therapy. Theranostics.

[117] Matsumoto K, Mizoue K, Kitamura K, Tse WC, Huber CP, Ishida T (1999). Structural basis of inhibition of cysteine proteases by E-64 and its derivatives. Biopolym - Pept Sci Sect.

[118] Bakar SN, Kue CS (2024). A Combination Therapy of Cyclophosphamide and Immunomodulating Agents in Cancer. Curr Cancer Drug Targets.

[119] Langeh U, Kumar V, Singh C, Singh A (2022). Drug-herb combination therapy in cancer management. Mol Biol Rep.

[120] Sharma A, Sharma L, Nandy SK, Payal N, Yadav S, Vargas-De-La-Cruz C (2023). Molecular Aspects and Therapeutic Implications of Herbal Compounds Targeting Different Types of Cancer. Molecules.

[121] Zhou H, Zhang M, Cao H, Du X, Zhang X, Wang J (2023). Research Progress on the Synergistic Anti-Tumor Effect of Natural Anti-Tumor Components of Chinese Herbal Medicine Combined with Chemotherapy Drugs. Pharmaceuticals (Basel).

[122] Jenča A, Mills DK, Ghasemi H, Saberian E, Forood AMK, Petrášová A (2024). Herbal Therapies for Cancer Treatment: A Review of Phytotherapeutic Efficacy. Biologics.

[123] Hong H, Baatar D, Hwang SG (2021). Anticancer Activities of Ginsenosides, the Main Active Components of Ginseng. Evid Based Complement Alternat Med.

[124] Ni B, Song X, Shi B, Wang J, Sun Q, Wang X (2022). Research progress of ginseng in the treatment of gastrointestinal cancers. Front Pharmacol.

[125] Fan M, Shan M, Lan X, Fang X, Song D, Luo H (2022). Anti-cancer effect and potential microRNAs targets of ginsenosides against breast cancer. Front Pharmacol.

[126] Li M, Wang X, Wang Y, Bao S, Chang Q, Liu L (2021). Strategies for Remodeling the Tumor Microenvironment Using Active Ingredients of Ginseng-A Promising Approach for Cancer Therapy. Front Pharmacol.

[127] Valdés-González JA, Sánchez M, Moratilla-Rivera I, Iglesias I, Gómez-Serranillos MP (2023). Immunomodulatory, Anti-Inflammatory, and Anti-Cancer Properties of Ginseng: A Pharmacological Update. Molecules.

[128] Liang Y, Zhao S (2008). Progress in understanding of ginsenoside biosynthesis. Plant Biol (Stuttg).

[129] Tanko AI, Hosawi S, Moglad E, Afzal M, Ghaboura N, Alzareaa SI (2025). Ginsenoside R_g3_ in Cancer Research: Current Trends and Future Prospects - A Review. Curr Med Chem.

[130] Wang J, Zeng L, Zhang Y, Qi W, Wang Z, Tian L (2022). Pharmacological properties, molecular mechanisms and therapeutic potential of ginsenoside R_g3_ as an antioxidant and anti-inflammatory agent. Front Pharmacol.

[131] Xiaodan S, Ying C (2022). Role of ginsenoside Rh2 in tumor therapy and tumor microenvironment immunomodulation. Biomed Pharmacother.

[132] Guan W, Qi W (2023). Ginsenoside Rh2: A shining and potential natural product in the treatment of human nonmalignant and malignant diseases in the near future. Phytomedicine.

[133] Han Q, Han L, Tie F, Wang Z, Ma C, Li J (2020). (20S)-Protopanaxadiol Ginsenosides Induced Cytotoxicity via Blockade of Autophagic Flux in HGC-27 Cells. Chem Biodivers.

[134] Chen F, Deng Z, Xiong Z, Zhang B, Yang J, Hu J (2015). A ROS-mediated lysosomal-mitochondrial pathway is induced by ginsenoside Rh2 in hepatoma HepG2 cells. Food Funct.

[135] Park HH, Choi SW, Lee GJ, Kim YD, Noh HJ, Oh SJ (2019). A formulated red ginseng extract inhibits autophagic flux and sensitizes to doxorubicin-induced cell death. J Ginseng Res.

[136] Oh JM, Kim E, Chun S (2019). Ginsenoside Compound K Induces Ros-Mediated Apoptosis and Autophagic Inhibition in Human Neuroblastoma Cells In Vitro and In Vivo. Int J Mol Sci.

[137] Kim DG, Jung KH, Lee DG, Yoon JH, Choi KS, Kwon SW (2014). 20(S)-Ginsenoside R_g3_ is a novel inhibitor of autophagy and sensitizes hepatocellular carcinoma to doxorubicin. Oncotarget.

[138] Wang XJ, Zhou RJ, Zhang N, Jing Z (2019). 20(S)-ginsenoside R_g3_ sensitizes human non-small cell lung cancer cells to icotinib through inhibition of autophagy. Eur J Pharmacol.

[139] Zhu Y, Liang J, Gao C, Wang A, Xia J, Hong C (2021). Multifunctional ginsenoside R_g3_-based liposomes for glioma targeting therapy. J Control Release.

[140] Li H, Huang N, Zhu W, Wu J, Yang X, Teng W (2018). Modulation the crosstalk between tumor-associated macrophages and non-small cell lung cancer to inhibit tumor migration and invasion by ginsenoside Rh2. BMC Cancer.

[141] Hong C, Liang J, Xia J, Zhu Y, Guo Y, Wang A (2020). One Stone Four Birds: A Novel Liposomal Delivery System Multi-functionalized with Ginsenoside Rh2 for Tumor Targeting Therapy. Nano-micro Lett.

[142] Chen L, Meng Y, Sun Q, Zhang Z, Guo X, Sheng X (2016). Ginsenoside compound K sensitizes human colon cancer cells to TRAIL-induced apoptosis via autophagy-dependent and -independent DR5 upregulation. Cell Death Dis.

[143] Hong C, Wang D, Liang J, Guo Y, Zhu Y, Xia J (2019). Novel ginsenoside-based multifunctional liposomal delivery system for combination therapy of gastric cancer. Theranostics.

[144] Yu H, Teng L, Meng Q, Li Y, Sun X, Lu J (2013). Development of liposomal ginsenoside Rg3: Formulation optimization and evaluation of its anticancer effects. Int J Pharm.

[145] Won HJ, Kim H Il, Park T, Kim H, Jo K, Jeon H (2019). Non-clinical pharmacokinetic behavior of ginsenosides. J Ginseng Res.

[146] Zheng S wen, Xiao S yuan, Wang J, Hou W, Wang Y ping (2019). Inhibitory Effects of Ginsenoside Ro on the Growth of B16F10 Melanoma via Its Metabolites. Molecules.

[147] Xu HL, Chen GH, Wu YT, Xie LP, Tan Z Bin, Liu B (2022). Ginsenoside Ro, an oleanolic saponin of Panax ginseng, exerts anti-inflammatory effect by direct inhibiting toll like receptor 4 signaling pathway. J Ginseng Res.

[148] Heid ME, Keyel PA, Kamga C, Shiva S, Watkins SC, Salter RD (2013). Mitochondrial reactive oxygen species induces NLRP3-dependent lysosomal damage and inflammasome activation. J Immunol.

[149] Song SB, Hwang ES (2020). High Levels of ROS Impair Lysosomal Acidity and Autophagy Flux in Glucose-Deprived Fibroblasts by Activating ATM and Erk Pathways. Biomolecules.

[150] Kawai A, Uchiyama H, Takano S, Nakamura N, Ohkuma S (2007). Autophagosome-lysosome fusion depends on the pH in acidic compartments in CHO cells. Autophagy.

[151] Wang YZ, Xu Q, Wu W, Liu Y, Jiang Y, Cai QQ (2018). Brain Transport Profiles of Ginsenoside Rb1 by Glucose Transporter 1: In Vitro and in Vivo. Front Pharmacol.

[152] Yuan Q, Liu H, Song C, Tian Y, Hassan W, Shabbir R (2025). Prevalence of GLUT1 overexpression in human cancers a systematic review and meta analysis. Discov Oncol.

[153] Peng Z, Wu WW, Yi P (2021). The Efficacy of Ginsenoside R_g3_ Combined with First-line Chemotherapy in the Treatment of Advanced Non-Small Cell Lung Cancer in China: A Systematic Review and Meta-Analysis of Randomized Clinical Trials. Front Pharmacol.

[154] Liu J, Wang Y, Yu Z, Lv G, Huang X, Lin H (2022). Functional Mechanism of Ginsenoside Compound K on Tumor Growth and Metastasis. Integr Cancer Ther.

[155] Liu T, Zhu L, Wang L (2022). A narrative review of the pharmacology of ginsenoside compound K. Ann Transl Med.

[156] Zhang R, Liao Y, Gao Y, Tian H, Wu S, Zeng Q (2024). Evaluation of the Efficacy, Safety, and Clinical Outcomes of Ginsenosides as Adjuvant Therapy in Hepatocellular Carcinoma: A Meta-Analysis and Systematic Review. Integr Cancer Ther.

[157] De Martino M, Daviaud C, Vanpouille-Box C (2021). Radiotherapy: An immune response modifier for immuno-oncology. Semin Immunol.

[158] Grassberger C, Ellsworth SG, Wilks MQ, Keane FK, Loeffler JS (2019). Assessing the interactions between radiotherapy and antitumour immunity. Nat Rev Clin Oncol.

[159] Roy A, Bera S, Saso L, Dwarakanath BS (2022). Role of autophagy in tumor response to radiation: Implications for improving radiotherapy. Front Oncol.

[160] Chandra RA, Keane FK, Voncken FEM, Thomas CR (2021). Contemporary radiotherapy: present and future. Lancet.

[161] Chow JCL, Ruda HE (2024). Mechanisms of Action in FLASH Radiotherapy: A Comprehensive Review of Physicochemical and Biological Processes on Cancerous and Normal Cells. Cells.

[162] Wu Y, Song Y, Wang R, Wang T (2023). Molecular mechanisms of tumor resistance to radiotherapy. Mol Cancer 2023 221.

[163] Morris ZS, Guy EI, Francis DM, Gressett MM, Werner LR, Carmichael LL (2016). In situ tumor vaccination by combining local radiation and tumor-specific antibody or immunocytokine treatments. Cancer Res.

[164] Vanpouille-Box C, Pilones KA, Wennerberg E, Formenti SC, Demaria S (2015). In situ vaccination by radiotherapy to improve responses to anti-CTLA-4 treatment. Vaccine.

[165] Gaipl US, Multhoff G, Scheithauer H, Lauber K, Hehlgans S, Frey B (2014). Kill and spread the word: Stimulation of antitumor immune responses in the context of radiotherapy. Immunotherapy.

[166] Yang H, Chen K, Meng Y, Chen Z, Xu Y, Zhou D (2025). Review: radiotherapy-mediated B cells within the TLS influence the tumor microenvironment. J Immunother Cancer.

[167] Dai D, Li X, Zhuang H, Ling Y, Chen L, Long C (2025). Landscape of the Peripheral Immune Response Induced by Intraoperative Radiotherapy Combined with Surgery in Early Breast Cancer Patients. Adv Sci.

[168] Sezen D, Barsoumian HB, He K, Hu Y, Wang Q, Abana CO (2022). Pulsed radiotherapy to mitigate high tumor burden and generate immune memory. Front Immunol.

[169] Craig DJ, Nanavaty NS, Devanaboyina M, Stanbery L, Hamouda D, Edelman G (2021). The abscopal effect of radiation therapy. Futur Oncol.

[170] Wang C, Han L, Zhang J, Ji Q, Guo X, Li Y (2025). Radiotherapy in combination with PD-1 and TIGIT blockade mediate antitumor abscopal effects and immune memory via CD8+ T cells. Cancer Lett.

[171] Abuodeh Y, Venkat P, Kim S (2016). Systematic review of case reports on the abscopal effect. Curr Probl Cancer.

[172] Xu J, Escamilla J, Mok S, David J, Priceman S, West B (2013). CSF1R signaling blockade stanches tumor-infiltrating myeloid cells and improves the efficacy of radiotherapy in prostate cancer. Cancer Res.

[173] Li B, Tan Y, Lei JH, Deng M, Yu X, Wang X (2025). Alkaline Adjuvant Regulates Proteolytic Activity of Macrophages for Antigen Cross-Presentation and Potentiates Radioimmunotherapy. Adv Mater.

[174] Zheng Z, Jia S, Shao C, Shi Y (2020). Irradiation induces cancer lung metastasis through activation of the cGAS-STING-CCL5 pathway in mesenchymal stromal cells. Cell Death Dis.

[175] Zhang X, Wang J, Li X, Wang D (2018). Lysosomes contribute to radioresistance in cancer. Cancer Lett.

[176] Liu H, Xiao Y, Dai C, Chen K, Xu X, Cai J Research Advances of the Autophagy-Regulated Radiosensitivity. Cell Prolif. 2025.

[177] Liu M, O'Connor RS, Trefely S, Graham K, Snyder NW, Beatty GL (2019). Metabolic rewiring of macrophages by CpG potentiates clearance of cancer cells and overcomes tumor-expressed CD47-mediated “don't-eat-me” signal. Nat Immunol.

[178] Holtmeier W, Holtmann G, Caspary WF, Weingärtner U (2007). On-demand treatment of acute heartburn with the antacid hydrotalcite compared with famotidine and placebo: randomized double-blind cross-over study. J Clin Gastroenterol.

[179] Jin W, Lee D, Jeon Y, Park DH (2020). Biocompatible Hydrotalcite Nanohybrids for Medical Functions. Miner 2020, Vol 10, Page 172.

[180] Simeonidis K, Kaprara E, Rivera-Gil P, Xu R, Teran FJ, Kokkinos E (2021). Hydrotalcite-embedded magnetite nanoparticles for hyperthermia-triggered chemotherapy. Nanomaterials.

[181] Liu H, Peng J, Huang L, Ruan D, Li Y, Yuan F (2023). The role of lysosomal peptidases in glioma immune escape: underlying mechanisms and therapeutic strategies. Front Immunol.

[182] Yalamarty SSK, Filipczak N, Li X, Subhan MA, Parveen F, Ataide JA (2023). Mechanisms of Resistance and Current Treatment Options for Glioblastoma Multiforme (GBM). Cancers (Basel).

[183] Vilar JB, Christmann M, Tomicic MT (2022). Alterations in Molecular Profiles Affecting Glioblastoma Resistance to Radiochemotherapy: Where Does the Good Go?. Cancers (Basel).

[184] Zhang X, Xu R, Zhang C, Xu Y, Han M, Huang B (2017). Trifluoperazine, a novel autophagy inhibitor, increases radiosensitivity in glioblastoma by impairing homologous recombination. J Exp Clin Cancer Res.

[185] Howland RH (2016). Trifluoperazine: A sprightly old drug. J Psychosoc Nurs Ment Health Serv.

[186] Kang S, Hong J, Lee JM, Moon HE, Jeon B, Choi J (2017). Trifluoperazine, a well-known antipsychotic, inhibits glioblastoma invasion by binding to calmodulin and disinhibiting calcium release channel IP3R. Mol Cancer Ther.

[187] Domańska U, Pelczarska A, Pobudkowska A (2011). Solubility and pK a determination of six structurally related phenothiazines. Int J Pharm.

[188] Vater M, Möckl L, Gormanns V, Schultz Fademrecht C, Mallmann AM, Ziegart-Sadowska K (2017). New insights into the intracellular distribution pattern of cationic amphiphilic drugs. Sci Rep.

[189] Zhang X, Ding K, Ji J, Parajuli H, Aasen SN, Espedal H (2020). Trifluoperazine prolongs the survival of experimental brain metastases by STAT3-dependent lysosomal membrane permeabilization. Am J Cancer Res.

[190] Xu Q, Zhang H, Liu H, Han Y, Qiu W, Li Z (2022). Inhibiting autophagy flux and DNA repair of tumor cells to boost radiotherapy of orthotopic glioblastoma. Biomaterials.

[191] Thomé R, Lopes SCP, Costa FTM, Verinaud L (2013). Chloroquine: Modes of action of an undervalued drug. Immunol Lett.

[192] De Sanctis JB, Charris J, Blanco Z, Ramírez H, Martínez GP, Mijares MR (2022). Molecular Mechanisms of Chloroquine and Hydroxychloroquine Used in Cancer Therapy. Anticancer Agents Med Chem.

[193] Ferreira PMP, Sousa RWR de, Ferreira JR de O, Militão GCG, Bezerra DP (2021). Chloroquine and hydroxychloroquine in antitumor therapies based on autophagy-related mechanisms. Pharmacol Res.

[194] Mauthe M, Orhon I, Rocchi C, Zhou X, Luhr M, Hijlkema KJ (2018). Chloroquine inhibits autophagic flux by decreasing autophagosome-lysosome fusion. Autophagy.

[195] Natsume A, Kinjo S, Yuki K, Kato T, Ohno M, Motomura K (2011). Glioma-initiating cells and molecular pathology: Implications for therapy. Brain Tumor Pathol.

[196] Bao S, Wu Q, McLendon RE, Hao Y, Shi Q, Hjelmeland AB (2006). Glioma stem cells promote radioresistance by preferential activation of the DNA damage response. Nature.

[197] Brown CE, Starr R, Aguilar B, Shami AF, Martinez C, D'Apuzzo M (2012). Stem-like tumor-initiating cells isolated from IL13Rα2 expressing gliomas are targeted and killed by IL13-zetakine-redirected T cells. Clin Cancer Res.

[198] Zhuang W, Long L, Zheng B, Ji W, Yang N, Zhang Q (2012). Curcumin promotes differentiation of glioma-initiating cells by inducing autophagy. Cancer Sci.

[199] Tao Z, Li T, Ma H, Yang Y, Zhang C, Hai L (2018). Autophagy suppresses self-renewal ability and tumorigenicity of glioma-initiating cells and promotes Notch1 degradation. Cell Death Dis.

[200] Zhuang W, Li B, Long L, Chen L, Huang Q, Liang Z (2011). Induction of autophagy promotes differentiation of glioma-initiating cells and their radiosensitivity. Int J Cancer.

[201] Ye H, Chen M, Cao F, Huang H, Zhan R, Zheng X (2016). Chloroquine, an autophagy inhibitor, potentiates the radiosensitivity of glioma initiating cells by inhibiting autophagy and activating apoptosis. BMC Neurol.

[202] Buccarelli M, Marconi M, Pacioni S, De Pasqualis I, D'Alessandris QG, Martini M (2018). Inhibition of autophagy increases susceptibility of glioblastoma stem cells to temozolomide by igniting ferroptosis. Cell Death Dis.

[203] Li C, Liu Y, Liu H, Zhang W, Shen C, Cho K (2015). Impact of autophagy inhibition at different stages on cytotoxic effect of autophagy inducer in glioblastoma cells. Cell Physiol Biochem.

[204] Briceño E, Reyes S, Sotelo J (2003). Therapy of glioblastoma multiforme improved by the antimutagenic chloroquine. Neurosurg Focus.

[205] Sotelo J, Briceño E, López-González MA (2006). Adding chloroquine to conventional treatment for glioblastoma multiforme: A randomized, double-blind, placebo-controlled trial. Ann Intern Med.

[206] Rojas-Puentes LL, Gonzalez-Pinedo M, Crismatt A, Ortega-Gomez A, Gamboa-Vignolle C, Nuñez-Gomez R (2013). Phase II randomized, double-blind, placebo-controlled study of whole-brain irradiation with concomitant chloroquine for brain metastases. Radiat Oncol.

[207] Bilger A, Bittner MI, Grosu AL, Wiedenmann N, Meyer PT, Firat E (2014). FET-PET-based reirradiation and chloroquine in patients with recurrent glioblastoma: First tolerability and feasibility results. Strahlentherapie und Onkol.

[208] Compter I, Eekers DBP, Hoeben A, Rouschop KMA, Reymen B, Ackermans L (2021). Chloroquine combined with concurrent radiotherapy and temozolomide for newly diagnosed glioblastoma: a phase IB trial. Autophagy.

[209] Witte HM, Riecke A, Steinestel K, Schulz C, Küchler J, Gebauer N (2024). The addition of chloroquine and bevacizumab to standard radiochemotherapy for recurrent glioblastoma multiforme. Br J Neurosurg.

[210] Anand K, Niravath P, Patel T, Ensor J, Rodriguez A, Boone T (2021). A Phase II Study of the Efficacy and Safety of Chloroquine in Combination With Taxanes in the Treatment of Patients With Advanced or Metastatic Anthracycline-refractory Breast Cancer. Clin Breast Cancer.

[211] Samaras P, Tusup M, Nguyen-Kim TDL, Seifert B, Bachmann H, von Moos R (2017). Phase I study of a chloroquine-gemcitabine combination in patients with metastatic or unresectable pancreatic cancer. Cancer Chemother Pharmacol.

[212] Eldredge HB, DeNittis A, DuHadaway JB, Chernick M, Metz R, Prendergast GC (2013). Concurrent whole brain radiotherapy and short-course chloroquine in patients with brain metastases: A pilot trial. J Radiat Oncol.

[213] Stevens DM, Crist RM, Stern ST (2020). Nanomedicine Reformulation of Chloroquine and Hydroxychloroquine. Molecules.

[214] Krajcer A, Grzywna E, Lewandowska-Łańcucka J (2023). Strategies increasing the effectiveness of temozolomide at various levels of anti-GBL therapy. Biomed Pharmacother.

[215] Jezierzański M, Nafalska N, Stopyra M, Furgoł T, Miciak M, Kabut J (2024). Temozolomide (TMZ) in the Treatment of Glioblastoma Multiforme—A Literature Review and Clinical Outcomes. Curr Oncol.

[216] Gerber DE, Grossman SA, Zeltzman M, Parisi MA, Kleinberg L (2007). The impact of thrombocytopenia from temozolomide and radiation in newly diagnosed adults with high-grade gliomas. Neuro Oncol.

[217] Lee SW, Kim HK, Lee NH, Yi HY, Kim HS, Hong SH (2015). The synergistic effect of combination temozolomide and chloroquine treatment is dependent on autophagy formation and p53 status in glioma cells. Cancer Lett.

[218] Hsu SPC, Kuo JS, Chiang HC, Wang HE, Wang YS, Huang CC (2018). Temozolomide, sirolimus and chloroquine is a new therapeutic combination that synergizes to disrupt lysosomal function and cholesterol homeostasis in GBM cells. Oncotarget.

[219] Alyassin Y, Sayed EG, Mehta P, Ruparelia K, Arshad MS, Rasekh M (2020). Application of mesoporous silica nanoparticles as drug delivery carriers for chemotherapeutic agents. Drug Discov Today.

[220] Hu J, Wang Q, Wang Y, You G, Li P, Zhao L (2020). Polydopamine-based surface modification of hemoglobin particles for stability enhancement of oxygen carriers. J Colloid Interface Sci.

[221] Wu H, Wei M, Xu Y, Li Y, Zhai X, Su P (2022). PDA-Based Drug Delivery Nanosystems: A Potential Approach for Glioma Treatment. Int J Nanomedicine.

[222] Zhou C, Yang Q, Zhou X, Jia N (2022). PDA-coated CPT@MIL-53(Fe)-based theranostic nanoplatform for pH-responsive and MRI-guided chemotherapy. J Mater Chem B.

[223] D'Souza SE, Ginsberg MH, Plow EF (1991). Arginyl-glycyl-aspartic acid (RGD): a cell adhesion motif. Trends Biochem Sci.

[224] Javid H, Oryani MA, Rezagholinejad N, Esparham A, Tajaldini M, Karimi-Shahri M (2024). RGD peptide in cancer targeting: Benefits, challenges, solutions, and possible integrin-RGD interactions. Cancer Med.

[225] Schottelius M, Laufer B, Kessler H, Wester HJ (2009). Ligands for mapping αvβ3-integrin expression in vivo. Acc Chem Res.

[226] Liu S (2009). Radiolabeled cyclic RGD peptides as integrin αvβ 3-targeted radiotracers: Maximizing binding affinity via bivalency. Bioconjug Chem.

[227] Kesharwani P, Chandra J, Karim S, Gupta G, Karwasra R, Sharma A (2024). αvβ3 integrin targeting RGD peptide-based nanoparticles as an effective strategy for selective drug delivery to tumor microenvironment. J Drug Deliv Sci Technol.

[228] Demircioglu F, Hodivala-Dilke K (2016). αvβ3 Integrin and tumour blood vessels — learning from the past to shape the future. Curr Opin Cell Biol.

[229] Liu Y, Yang Y, Zhang C (2013). A concise review of magnetic resonance molecular imaging of tumor angiogenesis by targeting integrin αvβ3 with magnetic probes. Int J Nanomedicine.

[230] Beer AJ, Kessler H, Wester HJ, Schwaiger M (2011). PET Imaging of Integrin αVβ3 Expression. Theranostics.

[231] Beer AJ, Schwaiger M (2008). Imaging of integrin αvβ3 expression. Cancer Metastasis Rev.

[232] de Vries LH, Lodewijk L, Pijnappel EW, van Diest PJ, Schepers A, Bonenkamp HJ (2024). Expression of integrin αvβ3 in medullary thyroid carcinoma. Futur Oncol.

[233] Liu Y, He JX, Ji B, Wang JF, Zhang L, Pang ZQ (2023). Comprehensive analysis of integrin αvβ3/α6β1 in prognosis and immune escape of prostate cancer. Aging (Albany NY).

[234] Trajkovic-Arsic M, Mohajerani P, Sarantopoulos A, Kalideris E, Steiger K, Esposito I (2014). Multimodal molecular imaging of integrin avb3 for in vivo detection of pancreatic cancer. J Nucl Med.

[235] Bello L, Francolini M, Marthyn P, Zhang J, Carroll RS, Nikas DC (2001). αvβ3 and αvβ5 integrin expression in glioma periphery. Neurosurgery.

[236] Zhang P, Cao F, Zhang J, Tan Y, Yao S (2023). Temozolomide and chloroquine co-loaded mesoporous silica nanoparticles are effective against glioma. Heliyon.

[237] Cui Y, Song X, Li S, He B, Yuan L, Dai W (2017). The impact of receptor recycling on the exocytosis of αvβ3 integrin targeted gold nanoparticles. Oncotarget.

[238] Peng J, Wang Q, Zhou J, Zhao S, Di P, Chen Y (2021). Targeted Lipid Nanoparticles Encapsulating Dihydroartemisinin and Chloroquine Phosphate for Suppressing the Proliferation and Liver Metastasis of Colorectal Cancer. Front Pharmacol.

[239] Maltsev O V, Marelli UK, Kapp TG, Di Leva FS, Di Maro S, Nieberler M (2016). Stable Peptides Instead of Stapled Peptides: Highly Potent αvβ6-Selective Integrin Ligands. Angew Chemie - Int Ed.

[240] Nieberler M, Reuning U, Reichart F, Notni J, Wester HJ, Schwaiger M (2017). Exploring the role of RGD-recognizing integrins in cancer. Cancers (Basel).

[241] Cantor D, Slapetova I, Kan A, McQuade LR, Baker MS (2013). Overexpression of αvβ6 integrin alters the colorectal cancer cell proteome in favor of elevated proliferation and a switching in cellular adhesion that increases invasion. J Proteome Res.

[242] Peng C, Zou X, Xia W, Gao H, Li Z, Liu N (2018). Integrin αvβ6 plays a bi-directional regulation role between colon cancer cells and cancer-associated fibroblasts. Biosci Rep.

[243] Cantor DI, Cheruku HR, Nice EC, Baker MS (2015). Integrin αvβ6 sets the stage for colorectal cancer metastasis. Cancer Metastasis Rev.

[244] Busenhart P, Montalban-Arques A, Katkeviciute E, Morsy Y, Van Passen C, Hering L (2022). Inhibition of integrin αvβ6 sparks T-cell antitumor response and enhances immune checkpoint blockade therapy in colorectal cancer. J Immunother Cancer.

[245] Bhaw-Luximon A, Jhurry D (2017). Artemisinin and its derivatives in cancer therapy: Status of progress, mechanism of action, and future perspectives. Cancer Chemother Pharmacol.

[246] Li Q, Ma Q, Cheng J, Zhou X, Pu W, Zhong X (2021). Dihydroartemisinin as a sensitizing agent in cancer therapies. Onco Targets Ther.

[247] Yu R, Jin G, Fujimoto M (2021). Dihydroartemisinin: A Potential Drug for the Treatment of Malignancies and Inflammatory Diseases. Front Oncol.

[248] Dong L, He J, Luo L, Wang K (2023). Targeting the Interplay of Autophagy and ROS for Cancer Therapy: An Updated Overview on Phytochemicals. Pharmaceuticals.

[249] Shi X, Wang L, Ren L, Li J, Li S, Cui Q (2019). Dihydroartemisinin, an antimalarial drug, induces absent in melanoma 2 inflammasome activation and autophagy in human hepatocellular carcinoma HepG2215 cells. Phyther Res.

[250] Ma Q, Liao H, Xu L, Li Q, Zou J, Sun R (2020). Autophagy-dependent cell cycle arrest in esophageal cancer cells exposed to dihydroartemisinin. Chinese Med (United Kingdom).

[251] Rani K, Chand Sahu R, Chaudhuri A, Kumar DN, Arora S, Kumar D (2025). Exploring combinations of dihydroartemisinin for cancer therapy: A comprehensive review. Biochem Biophys Res Commun.

[252] Ganguli A, Choudhury D, Datta S, Bhattacharya S, Chakrabarti G (2014). Inhibition of autophagy by chloroquine potentiates synergistically anti-cancer property of artemisinin by promoting ROS dependent apoptosis. Biochimie.

[253] Tang T, Xia Q, Xi M (2021). Dihydroartemisinin and its anticancer activity against endometrial carcinoma and cervical cancer: Involvement of apoptosis, autophagy and transferrin receptor. Singapore Med J.

[254] Gupta N, Yelamanchi R (2021). Pancreatic adenocarcinoma: A review of recent paradigms and advances in epidemiology, clinical diagnosis and management. World J Gastroenterol.

[255] Mosalem OM, Abdelhakeem A, Abdel-Razeq NH, Babiker H (2025). Pancreatic ductal adenocarcinoma (PDAC): clinical progress in the last five years. Expert Opin Investig Drugs.

[256] Ray A, Callaway MK, Rodríguez-Merced NJ, Crampton AL, Carlson M, Emme KB (2022). Stromal architecture directs early dissemination in pancreatic ductal adenocarcinoma. JCI Insight.

[257] Rudnick JA, Debnath J (2021). Autophagy in host stromal fibroblasts supports tumor desmoplasia. Autophagy.

[258] Sandha KK, Kaur S, Sharma K, Ali SM, Ramajayan P, Kumar A (2025). Autophagy inhibition alleviates tumor desmoplasia and improves the efficacy of locally and systemically administered liposomal doxorubicin. J Control Release.

[259] Rudnick JA, Monkkonen T, Mar FA, Barnes JM, Starobinets H, Goldsmith J (2021). Autophagy in stromal fibroblasts promotes tumor desmoplasia and mammary tumorigenesis. Genes Dev.

[260] Zhou Y, Ma Y, Sheng J, Ma Y, Ding J, Zhou W (2024). Breaking Down Barriers in Drug Delivery by Stromal Remodeling Approaches in Pancreatic Cancer. Mol Pharm.

[261] Hilmi M, Delaye M, Muzzolini M, Nicolle R, Cros J, Hammel P (2023). The immunological landscape in pancreatic ductal adenocarcinoma and overcoming resistance to immunotherapy. Lancet Gastroenterol Hepatol.

[262] Lu T, Prakash J (2021). Nanomedicine strategies to enhance tumor drug penetration in pancreatic cancer. Int J Nanomedicine.

[263] Gu X, Minko T (2024). Targeted Nanoparticle-Based Diagnostic and Treatment Options for Pancreatic Cancer. Cancers (Basel).

[264] Lian Y, Zeng S, Wen S, Zhao X, Fang C, Zeng N (2023). Review and Application of Integrin Alpha v Beta 6 in the Diagnosis and Treatment of Cholangiocarcinoma and Pancreatic Ductal Adenocarcinoma. Technol Cancer Res Treat.

[265] Hosotani R, Kawaguchi M, Masui T, Koshiba T, Ida J, Fujimoto K (1995). Expression of integrin αvβ3 in pancreatic carcinoma: Relation to mmp-2 activation and lymph node metastasis. Nursing (Lond).

[266] Halcrow PW, Geiger JD, Chen X (2021). Overcoming Chemoresistance: Altering pH of Cellular Compartments by Chloroquine and Hydroxychloroquine. Front Cell Dev Biol.

[267] McChesney EW (1983). Animal toxicity and pharmacokinetics of hydroxychloroquine sulfate. Am J Med.

[268] Cabral RT de S, Klumb EM, Couto MINN, Carneiro S (2019). Evaluation of toxic retinopathy caused by antimalarial medications with spectral domain optical coherence tomography. Arq Bras Oftalmol.

[269] Zeh HJ, Bahary N, Boone BA, Singhi AD, Miller-Ocuin JL, Normolle DP (2020). A Randomized Phase II Preoperative Study of Autophagy Inhibition With High-Dose Hydroxychloroquine and Gemcitabine/Nab-Paclitaxel in Pancreatic Cancer Patients. Clin Cancer Res.

[270] AlMasri SS, Zenati MS, Desilva A, Nassour I, Boone BA, Singhi AD (2021). Encouraging long-term survival following autophagy inhibition using neoadjuvant hydroxychloroquine and gemcitabine for high-risk patients with resectable pancreatic carcinoma. Cancer Med.

[271] Karasic TB, O'Hara MH, Loaiza-Bonilla A, Reiss KA, Teitelbaum UR, Borazanci E (2019). Effect of Gemcitabine and nab-Paclitaxel With or Without Hydroxychloroquine on Patients With Advanced Pancreatic Cancer: A Phase 2 Randomized Clinical Trial. JAMA Oncol.

[272] Wolpin BM, Rubinson DA, Wang X, Chan JA, Cleary JM, Enzinger PC (2014). Phase II and pharmacodynamic study of autophagy inhibition using hydroxychloroquine in patients with metastatic pancreatic adenocarcinoma. Oncologist.

[273] Boone BA, Zeh HJ, Bahary N (2018). Autophagy Inhibition in Pancreatic Adenocarcinoma. Clin Colorectal Cancer.

[274] McCubrey JA, Abrams SL, Follo MY, Manzoli L, Ratti S, Martelli AM (2023). Effects of chloroquine and hydroxychloroquine on the sensitivity of pancreatic cancer cells to targeted therapies. Adv Biol Regul.

[275] Boone BA, Bahary N, Zureikat AH, Moser AJ, Normolle DP, Wu WC (2015). Safety and Biologic Response of Pre-operative Autophagy Inhibition in Combination with Gemcitabine in Patients with Pancreatic Adenocarcinoma. Ann Surg Oncol.

[276] Chen X, Yu Q, Liu Y, Sheng Q, Shi K, Wang Y (2019). Synergistic cytotoxicity and co-autophagy inhibition in pancreatic tumor cells and cancer-associated fibroblasts by dual functional peptide-modified liposomes. Acta Biomater.

[277] Shi K, Li J, Cao Z, Yang P, Qiu Y, Yang B (2015). A pH-responsive cell-penetrating peptide-modified liposomes with active recognizing of integrin αvβ3 for the treatment of melanoma. J Control Release.

[278] Moreno-Vargas LM, Prada-Gracia D (2024). Cancer-Targeting Applications of Cell-Penetrating Peptides. Int J Mol Sci.

[279] Henriques ST, Costa J, Castanho MARB (2005). Translocation of β-galactosidase mediated by the cell-penetrating peptide pep-1 into lipid vesicles and human HeLa cells is driven by membrane electrostatic potential. Biochemistry.

[280] Wu Z, Huang D, Wang J, Zhao Y, Sun W, Shen X (2024). Engineering Heterogeneous Tumor Models for Biomedical Applications. Adv Sci.

[281] Hendifar AE, Petzel MQ, Zimmers TA, Denlinger CS, Matrisian LM, Picozzi VJ (2018). Pancreas Cancer-Associated Weight Loss. Oncologist.

[282] Wang Y, Tai X, Zhang L, Liu Y, Gao H, Chen J (2015). A novel antitumour strategy using bidirectional autophagic vesicles accumulation via initiative induction and the terminal restraint of autophagic flux. J Control Release.

[283] Shoji-Kawata S, Sumpter R, Leveno M, Campbell GR, Zou Z, Kinch L (2013). Identification of a candidate therapeutic autophagy-inducing peptide. Nature.

[284] McAfee Q, Zhang Z, Samanta A, Levi SM, Ma XH, Piao S (2012). Autophagy inhibitor Lys05 has single-agent antitumor activity and reproduces the phenotype of a genetic autophagy deficiency. Proc Natl Acad Sci U S A.

[285] Ebrahimi N, Fardi E, Ghaderi H, Palizdar S, Khorram R, Vafadar R (2023). Receptor tyrosine kinase inhibitors in cancer. Cell Mol Life Sci.

[286] Murugesan S, Murugesan J, Palaniappan S, Palaniappan S, Murugan T, Siddiqui SS (2021). Tyrosine Kinase Inhibitors (TKIs) in Lung Cancer Treatment: A Comprehensive Analysis. Curr Cancer Drug Targets.

[287] Liang X, Yang Q, Wu P, He C, Yin L, Xu F (2021). The synthesis review of the approved tyrosine kinase inhibitors for anticancer therapy in 2015-2020. Bioorg Chem.

[288] Wu D, Sun Q, Tang H, Xiao H, Luo J, Ouyang L (2025). Acquired resistance to tyrosine kinase targeted therapy: mechanism and tackling strategies. Drug Resist Updat.

[289] You Q, Li R, Yao J, Zhang YC, Sui X, Xiao CC (2024). Insights into lenvatinib resistance: mechanisms, potential biomarkers, and strategies to enhance sensitivity. Med Oncol.

[290] Du X, Yang B, An Q, Assaraf YG, Cao X, Xia J (2021). Acquired resistance to third-generation EGFR-TKIs and emerging next-generation EGFR inhibitors. Innovation.

[291] Zhitomirsky B, Assaraf YG (2016). Lysosomes as mediators of drug resistance in cancer. Drug Resist Updat.

[292] Ruzickova E, Skoupa N, Dolezel P, Smith DA, Mlejnek P (2019). The Lysosomal Sequestration of Tyrosine Kinase Inhibitors and Drug Resistance. Biomolecules.

[293] Calabretta B, Salomoni P (2011). Inhibition of autophagy: a new strategy to enhance sensitivity of chronic myeloid leukemia stem cells to tyrosine kinase inhibitors. Leuk Lymphoma.

[294] Abdel-Aziz AK, Abdel-Naim AB, Shouman S, Minucci S, Elgendy M (2017). From resistance to sensitivity: Insights and implications of biphasic modulation of autophagy by sunitinib. Front Pharmacol.

[295] Wang B, Lu D, Xuan M, Hu W (2017). Antitumor effect of sunitinib in human prostate cancer cells functions via autophagy. Exp Ther Med.

[296] Yu HC, Lin CS, Tai WT, Liu CY, Shiau CW, Chen KF (2013). Nilotinib induces autophagy in hepatocellular carcinoma through AMPK activation. J Biol Chem.

[297] Meng L, Zhao P, Hu Z, Ma W, Niu Y, Su J (2021). Nilotinib, A Tyrosine Kinase Inhibitor, Suppresses the Cell Growth and Triggers Autophagy in Papillary Thyroid Cancer. Anticancer Agents Med Chem.

[298] Elshazly AM, Xu J, Melhem N, Abdulnaby A, Elzahed AA, Saleh T (2024). Is Autophagy Targeting a Valid Adjuvant Strategy in Conjunction with Tyrosine Kinase Inhibitors?. Cancers (Basel).

[299] Tian Y, Lei Y, Fu Y, Sun H, Wang J, Xia F (2022). Molecular Mechanisms of Resistance to Tyrosine Kinase Inhibitors Associated with Hepatocellular Carcinoma. Curr Cancer Drug Targets.

[300] Gadducci A, Multinu F, Cosio S, Carinelli S, Ghioni M, Aletti GD (2021). Clear cell carcinoma of the ovary: Epidemiology, pathological and biological features, treatment options and clinical outcomes. Gynecol Oncol.

[301] Zhu C, Zhu J, Qian L, Liu H, Shen Z, Wu D (2021). Clinical characteristics and prognosis of ovarian clear cell carcinoma: a 10-year retrospective study. BMC Cancer.

[302] Heng DYC, Kollmannsberger C (2010). Sunitinib. Recent Results Cancer Res.

[303] AboulMagd AM, Abdelwahab NS (2021). Analysis of sunitinib malate, a multi-targeted tyrosine kinase inhibitor: A critical review. Microchem J.

[304] Anglesio MS, George J, Kulbe H, Friedlander M, Rischin D, Lemech C (2011). IL6-STAT3-HIF signaling and therapeutic response to the angiogenesis inhibitor sunitinib in ovarian clear cell cancer. Clin Cancer Res.

[305] Chan JK, Brady W, Monk BJ, Brown J, Shahin MS, Rose PG (2018). A phase II evaluation of sunitinib in the treatment of persistent or recurrent clear cell ovarian carcinoma: An NRG Oncology/Gynecologic Oncology Group Study (GOG-254). Gynecol Oncol.

[306] Gotink KJ, Broxterman HJ, Labots M, De Haas RR, Dekker H, Honeywell RJ (2011). Lysosomal sequestration of sunitinib: A novel mechanism of drug resistance. Clin Cancer Res.

[307] Zhitomirsky B, Assaraf YG (2015). Lysosomal sequestration of hydrophobic weak base chemotherapeutics triggers lysosomal biogenesis and lysosome-dependent cancer multidrug resistance. Oncotarget.

[308] Wu S, Huang L, Shen R, Bernard-Cacciarella M, Zhou P, Hu C (2020). Drug resistance-related sunitinib sequestration in autophagolysosomes of endothelial cells. Int J Oncol.

[309] Nowak-Sliwinska P, Weiss A, van Beijnum JR, Wong TJ, Kilarski WW, Szewczyk G (2015). Photoactivation of lysosomally sequestered sunitinib after angiostatic treatment causes vascular occlusion and enhances tumor growth inhibition. Cell Death Dis.

[310] Yamamoto S, Nakayama T, Seki H, Kawada C, Fukuhara H, Karashima T (2021). Sunitinib with photoirradiation-mediated reactive oxygen species generation induces apoptosis of renal cell carcinoma cells. Photodiagnosis Photodyn Ther.

[311] Abdel-Aziz AK, Shouman S, El-Demerdash E, Elgendy M, Abdel-Naim AB (2014). Chloroquine synergizes sunitinib cytotoxicity via modulating autophagic, apoptotic and angiogenic machineries. Chem Biol Interact.

[312] Wiedmer T, Blank A, Pantasis S, Normand L, Bill R, Krebs P (2017). Autophagy inhibition improves sunitinib efficacy in pancreatic neuroendocrine tumors via a lysosome-dependent mechanism. Mol Cancer Ther.

[313] Dyczynski M, Yu Y, Otrocka M, Parpal S, Braga T, Henley AB (2018). Targeting autophagy by small molecule inhibitors of vacuolar protein sorting 34 (Vps34) improves the sensitivity of breast cancer cells to Sunitinib. Cancer Lett.

[314] Patergnani S, Zampieri P, Bianchi N, Ippolito C, Gafà R, Lanza G (2025). Autophagy inhibition potentiates anti-cancer activity of Sunitinib in kidney cancer cells. Biol Direct.

[315] DeVorkin L, Hattersley M, Kim P, Ries J, Spowart J, Anglesio MS (2017). Autophagy Inhibition Enhances Sunitinib Efficacy in Clear Cell Ovarian Carcinoma. Mol Cancer Res.

[316] Amir M, Javed S (2021). A Review on the Therapeutic Role of TKIs in Case of CML in Combination With Epigenetic Drugs. Front Genet.

[317] Romero D (2024). Asciminib is safe and effective in patients with newly diagnosed CML. Nat Rev Clin Oncol.

[318] Patterson SD, Copland M (2023). The Bone Marrow Immune Microenvironment in CML: Treatment Responses, Treatment-Free Remission, and Therapeutic Vulnerabilities. Curr Hematol Malig Rep.

[319] Bhatia R (2019). TARGETING LEUKEMIA STEM CELL RESISTANCE IN CHRONIC MYELOGENOUS LEUKEMIA. Trans Am Clin Climatol Assoc.

[320] Al Hamad M (2022). Contribution of BCR-ABL molecular variants and leukemic stem cells in response and resistance to tyrosine kinase inhibitors: a review. F1000Research.

[321] Bellodi C, Lidonnici MR, Hamilton A, Helgason GV, Soliera AR, Ronchetti M (2009). Targeting autophagy potentiates tyrosine kinase inhibitor-induced cell death in Philadelphia chromosome-positive cells, including primary CML stem cells. J Clin Invest.

[322] Karvela M, Baquero P, Kuntz EM, Mukhopadhyay A, Mitchell R, Allan EK (2016). ATG7 regulates energy metabolism, differentiation and survival of Philadelphia-chromosome-positive cells. Autophagy.

[323] Baquero P, Dawson A, Mukhopadhyay A, Kuntz EM, Mitchell R, Olivares O (2019). Targeting quiescent leukemic stem cells using second generation autophagy inhibitors. Leukemia.

[324] DeRemer DL, Ustun C, Natarajan K (2008). Nilotinib: A second-generation tyrosine kinase inhibitor for the treatment of chronic myelogenous leukemia. Clin Ther.

[325] Kantarjian HM, Hughes TP, Larson RA, Kim DW, Issaragrisil S, le Coutre P (2021). Long-term outcomes with frontline nilotinib versus imatinib in newly diagnosed chronic myeloid leukemia in chronic phase: ENESTnd 10-year analysis. Leukemia.

[326] Rheinländer A, Schraven B, Bommhardt U (2018). CD45 in human physiology and clinical medicine. Immunol Lett.

[327] Hallek M (2025). Chronic Lymphocytic Leukemia: 2025 Update on the Epidemiology, Pathogenesis, Diagnosis, and Therapy. Am J Hematol.

[328] Siegel RL, Giaquinto AN, Jemal A (2024). Cancer statistics, 2024. CA Cancer J Clin.

[329] Schmid VK, Hobeika E (2024). B cell receptor signaling and associated pathways in the pathogenesis of chronic lymphocytic leukemia. Front Oncol.

[330] Iatrou A, Agathangelidis A, Bordini J, Stamatopoulos K, Ghia P Autonomous B-cell Receptor Signaling in Chronic Lymphocytic Leukemia. Hematol Oncol Clin North Am. 2025.

[331] Wang X, Kokabee L, Kokabee M, Conklin DS (2021). Bruton's Tyrosine Kinase and Its Isoforms in Cancer. Front cell Dev Biol.

[332] McDonald C, Xanthopoulos C, Kostareli E (2021). The role of Bruton's tyrosine kinase in the immune system and disease. Immunology.

[333] Patton JT, Woyach JA (2024). Targeting the B cell receptor signaling pathway in chronic lymphocytic leukemia. Semin Hematol.

[334] Yin S, Zheng X, Zhang W, Zhao H, Zhang R, Li W (2024). Efficacy and safety of new-generation Bruton tyrosine kinase inhibitors in chronic lymphocytic leukemia/small lymphocytic lymphoma: a systematic review and meta-analysis. Ann Hematol.

[335] Wang J, Liu X, Hong Y, Wang S, Chen P, Gu A (2017). Ibrutinib, a Bruton's tyrosine kinase inhibitor, exhibits antitumoral activity and induces autophagy in glioblastoma. J Exp Clin Cancer Res.

[336] Sun FD, Wang PC, Shang J, Zou SH, Du X (2018). Ibrutinib presents antitumor activity in skin cancer and induces autophagy. Eur Rev Med Pharmacol Sci.

[337] Timofeeva N, Gandhi V (2021). Ibrutinib combinations in CLL therapy: scientific rationale and clinical results. Blood Cancer J.

[338] Brown JR, Eichhorst B, Hillmen P, Jurczak W, Kaźmierczak M, Lamanna N (2023). Zanubrutinib or Ibrutinib in Relapsed or Refractory Chronic Lymphocytic Leukemia. N Engl J Med.

[339] Das S, Dielschneider R, Chanas-LaRue A, Johnston JB, Gibson SB (2018). Antimalarial drugs trigger lysosome-mediated cell death in chronic lymphocytic leukemia (CLL) cells. Leuk Res.

[340] Dielschneider RF, Eisenstat H, Mi S, Curtis JM, Xiao W, Johnston JB (2016). Lysosomotropic agents selectively target chronic lymphocytic leukemia cells due to altered sphingolipid metabolism. Leukemia.

[341] Manivannan MS, Yang X, Patel N, Peters A, Johnston JB, Gibson SB (2024). Lysosome-Disrupting Agents in Combination with Venetoclax Increase Apoptotic Response in Primary Chronic Lymphocytic Leukemia (CLL) Cells Mediated by Lysosomal Cathepsin D Release and Inhibition of Autophagy. Cells.

[342] Yang Z, Du Y, Lei L, Xia X, Wang X, Tong F (2023). Co-delivery of ibrutinib and hydroxychloroquine by albumin nanoparticles for enhanced chemotherapy of glioma. Int J Pharm.

[343] Chanas-Larue A, Villalpando-Rodriguez GE, Henson ES, Johnston JB, Gibson SB (2020). Antihistamines are synergistic with Bruton's tyrosine kinase inhibiter ibrutinib mediated by lysosome disruption in chronic lymphocytic leukemia (CLL) cells. Leuk Res.

[344] Trybus E, Trybus W (2024). H1 Antihistamines—Promising Candidates for Repurposing in the Context of the Development of New Therapeutic Approaches to Cancer Treatment. Cancers (Basel).

[345] Ellegaard AM, Dehlendorff C, Vind AC, Anand A, Cederkvist L, Petersen NHT (2016). Repurposing Cationic Amphiphilic Antihistamines for Cancer Treatment. EBioMedicine.

[346] Le Joncour V, Filppu P, Hyvönen M, Holopainen M, Turunen SP, Sihto H (2019). Vulnerability of invasive glioblastoma cells to lysosomal membrane destabilization. EMBO Mol Med.

[347] Cornet-Masana JM, Banús-Mulet A, Carbó JM, Torrente MÁ, Guijarro F, Cuesta-Casanovas L (2019). Dual lysosomal-mitochondrial targeting by antihistamines to eradicate leukaemic cells. EBioMedicine.

[348] Ahmad SS, Duke S, Jena R, Williams M V, Burnet NG (2012). Advances in radiotherapy. BMJ.

[349] Braunstein S, Nakamura JL (2013). Radiotherapy-Induced Malignancies: Review of Clinical Features, Pathobiology, and Evolving Approaches for Mitigating Risk. Front Oncol.

[350] Guo X, Osouli S, Shahripour RB (2023). Review of Cerebral Radiotherapy-Induced Vasculopathy in Pediatric and Adult Patients. Adv Biol.

[351] Koutroumpakis E, Deswal A, Yusuf SW, Abe J ichi, Nead KT, Potter AS (2022). Radiation-Induced Cardiovascular Disease: Mechanisms, Prevention, and Treatment. Curr Oncol Rep.

[352] Tödt K, Engström M, Ekström M, Efverman A (2022). Fatigue During Cancer-Related Radiotherapy and Associations with Activities, Work Ability and Quality of Life: Paying Attention to Subgroups more Likely to Experience Fatigue. Integr Cancer Ther.

[353] Park J, Lee YK, Park IK, Hwang SR (2021). Current limitations and recent progress in nanomedicine for clinically available photodynamic therapy. Biomedicines.

[354] Gunaydin G, Gedik ME, Ayan S (2021). Photodynamic Therapy—Current Limitations and Novel Approaches. Front Chem.

[355] Stolik S, Delgado JA, Pérez A, Anasagasti L (2000). Measurement of the penetration depths of red and near infrared light in human ″*ex vivo*″ tissues. J Photochem Photobiol B Biol.

[356] Henderson TA, Morries LD (2015). Near-infrared photonic energy penetration: can infrared phototherapy effectively reach the human brain?. Neuropsychiatr Dis Treat.

[357] Yang Y, Wang D, Wang B, Wang X (2025). Management of Adverse Reactions to Photodynamic Therapy. Photodyn Ther Dermatology.

[358] Hoogenboom M, Eikelenboom D, den Brok MH, Heerschap A, Fütterer JJ, Adema GJ (2015). Mechanical High-Intensity Focused Ultrasound Destruction of Soft Tissue: Working Mechanisms and Physiologic Effects. Ultrasound Med Biol.

[359] Lafond M, Yoshizawa S, Umemura S ichiro (2019). Sonodynamic Therapy: Advances and Challenges in Clinical Translation. J Ultrasound Med.

[360] Tao W, Lai Y, Zhou X, Yang G, Wu P, Yuan L (2025). A narrative review: Ultrasound-Assisted drug delivery: Improving treatments via multiple mechanisms. Ultrasonics.

[361] Delaney LJ, Isguven S, Eisenbrey JR, Hickok NJ, Forsberg F (2022). Making waves: how ultrasound-targeted drug delivery is changing pharmaceutical approaches. Mater Adv.

[362] E (2012). Konofagou E, Tunga YS, Choia J, Deffieuxa T, Baseria B, Vlachosa F. Ultrasound-Induced Blood-Brain Barrier Opening. Curr Pharm Biotechnol.

[363] Alonso A (2015). Ultrasound-induced blood-brain barrier opening for drug delivery. Front Neurol Neurosci.

[364] Deng Z, Sheng Z, Yan F (2019). Ultrasound-Induced Blood-Brain-Barrier Opening Enhances Anticancer Efficacy in the Treatment of Glioblastoma: Current Status and Future Prospects. J Oncol.

[365] Grasso G, Torregrossa F, Noto M, Bruno E, Feraco P, Buscemi F (2023). MR-guided focused ultrasound-induced blood-brain barrier opening for brain metastasis: a review. Neurosurg Focus.

[366] Collins VG, Hutton D, Hossain-Ibrahim K, Joseph J, Banerjee S (2025). The abscopal effects of sonodynamic therapy in cancer: Translational Therapeutics. Br J Cancer.

[367] Yang Y, Huang J, Liu M, Qiu Y, Chen Q, Zhao T (2023). Emerging Sonodynamic Therapy-Based Nanomedicines for Cancer Immunotherapy. Adv Sci.

[368] Du JR, Wang Y, Yue ZH, Zhang HY, Wang H, Sui GQ (2023). Recent advances in sonodynamic immunotherapy. J Cancer Res Clin Oncol.

[369] Wang T, Peng W, Du M, Chen Z (2023). Immunogenic sonodynamic therapy for inducing immunogenic cell death and activating antitumor immunity. Front Oncol.

[370] Liang S, Yao J, Liu D, Rao L, Chen X, Wang Z (2023). Harnessing Nanomaterials for Cancer Sonodynamic Immunotherapy. Adv Mater.

[371] Li K, Li L, Xie X, Zhu J, Xia D, Xiang L (2024). Spatially confined photoacoustic effects of responsive nanoassembly boosts lysosomal membrane permeabilization and immunotherapy of triple-negative breast cancer. Acta Biomater.

[372] Su X, Wang X, Liu Q, Wang P, Xu C, Leung AW (2015). The role of Beclin 1 in SDT-induced apoptosis and autophagy in human leukemia cells. Int J Radiat Biol.

[373] Wang X, Wang P, Zhang K, Su X, Hou J, Liu Q (2013). Initiation of autophagy and apoptosis by sonodynamic therapy in murine leukemia L1210 cells. Toxicol Vitr.

[374] Wang X, Liu Q, Wang Z, Wang P, Zhao P, Zhao X (2010). Role of autophagy in sonodynamic therapy-induced cytotoxicity in S180 cells. Ultrasound Med Biol.

[375] Su X, Wang P, Yang S, Zhang K, Liu Q, Wang X (2015). Sonodynamic therapy induces the interplay between apoptosis and autophagy in K562 cells through ROS. Int J Biochem Cell Biol.

[376] Zhou L, Huo M, Qian X, Ding L, Yu L, Feng W (2021). Autophagy blockade synergistically enhances nanosonosensitizer-enabled sonodynamic cancer nanotherapeutics. J Nanobiotechnology.

[377] Zhang Y, Zhao Y, Zhang Y, Liu Q, Zhang M, Tu K (2022). The crosstalk between sonodynamic therapy and autophagy in cancer. Front Pharmacol.

[378] Du X, Song J, Zhang Z, Liu J, Xu D (2023). Inhibition of autophagy promotes sonodynamic therapy-induced apoptosis of pancreatic cancer cells. Folia Histochem Cytobiol.

[379] Liu Y, Li B, Yang R, Shang C, Bai Y, Zheng B (2025). Ultrasound-triggered lysosomal alkalinization to block autophagy in tumor therapy. Biomaterials.

[380] Wu Y, Zou J, Tang K, Xia Y, Wang X, Song L (2024). From electricity to vitality: the emerging use of piezoelectric materials in tissue regeneration. Burn Trauma.

[381] Chen S, Tong X, Huo Y, Liu S, Yin Y, Tan ML (2024). Piezoelectric Biomaterials Inspired by Nature for Applications in Biomedicine and Nanotechnology. Adv Mater.

[382] Tijore A, Margadant F, Dwivedi N, Morgan L, Yao M, Hariharan A (2025). Ultrasound-mediated mechanical forces activate selective tumor cell apoptosis. Bioeng Transl Med.

[383] Manbachi A, Cobbold RSC (2011). Development and Application of Piezoelectric Materials for Ultrasound Generation and Detection. Ultrasound.

[384] Wu X, Liang J, Shu J, Li Z, Yin T, Zhang X (2025). Narrow-Bandgap Iridium(III)-C3N5 Nanocomplex as an Oxygen Self-Sufficient Piezo-Sonosensitizer for Hypoxic Tumor Sonodynamic Immunotherapy. J Am Chem Soc.

[385] Harris B, Saleem S, Cook N, Searle E (2022). Targeting hypoxia in solid and haematological malignancies. J Exp Clin Cancer Res.

[386] Fu T, Dai LJ, Wu SY, Xiao Y, Ma D, Jiang YZ (2021). Spatial architecture of the immune microenvironment orchestrates tumor immunity and therapeutic response. J Hematol Oncol.

[387] Xiao Y, Yu D (2021). Tumor microenvironment as a therapeutic target in cancer. Pharmacol Ther.

[388] Assaraf YG, Brozovic A, Gonçalves AC, Jurkovicova D, Linē A, Machuqueiro M (2019). The multi-factorial nature of clinical multidrug resistance in cancer. Drug Resist Updat.

[389] Tang T, Huang X, Zhang G, Hong Z, Bai X, Liang T (2021). Advantages of targeting the tumor immune microenvironment over blocking immune checkpoint in cancer immunotherapy. Signal Transduct Target Ther.

[390] Xu L, Zou C, Zhang S, Chu TSM, Zhang Y, Chen W (2022). Reshaping the systemic tumor immune environment (STIE) and tumor immune microenvironment (TIME) to enhance immunotherapy efficacy in solid tumors. J Hematol Oncol.

[391] Wu B, Shi X, Jiang M, Liu H (2023). Cross-talk between cancer stem cells and immune cells: potential therapeutic targets in the tumor immune microenvironment. Mol Cancer.

[392] Wolf B, Weydandt L, Dornhöfer N, Hiller GGR, Höhn AK, Nel I (2023). Desmoplasia in cervical cancer is associated with a more aggressive tumor phenotype. Sci Reports 2023 131.

[393] Mittal S, Brown NJ, Holen I (2018). The breast tumor microenvironment: role in cancer development, progression and response to therapy. Expert Rev Mol Diagn.

[394] Bremnes RM, Dønnem T, Al-Saad S, Al-Shibli K, Andersen S, Sirera R (2011). The role of tumor stroma in cancer progression and prognosis: Emphasis on carcinoma-associated fibroblasts and non-small cell lung cancer. J Thorac Oncol.

[395] Sun T, Hirakawa Y, Murad F, Schmults C, Piris A (2024). A Proposed Grading System for Desmoplasia in Cutaneous Squamous Cell Carcinoma Predicts Death from Disease. J Invest Dermatol.

[396] Laskaratos FM, Rombouts K, Caplin M, Toumpanakis C, Thirlwell C, Mandair D (2017). Neuroendocrine tumors and fibrosis: An unsolved mystery?. Cancer.

[397] Goudar VS, Koduri MP, Ta YNN, Chen Y, Chu LA, Lu LS (2021). Impact of a Desmoplastic Tumor Microenvironment for Colon Cancer Drug Sensitivity: A Study with 3D Chimeric Tumor Spheroids. ACS Appl Mater Interfaces.

[398] Hu Q, Wang Y, Yao S, Mao Y, Liu L, Li Z (2023). Desmoplastic Reaction Associates with Prognosis and Adjuvant Chemotherapy Response in Colorectal Cancer: A Multicenter Retrospective Study. Cancer Res Commun.

[399] Suzuki S, Nagakawa Y, Miyamoto R, Osakabe H, Kiya Y, Yamaguchi H (2024). Prognostic factors based on histological categorization of desmoplastic reactions in pancreatic cancer. World J Surg.

[400] Ghavami S, Cunnington RH, Gupta S, Yeganeh B, Filomeno KL, Freed DH (2015). Autophagy is a regulator of TGF-β1-induced fibrogenesis in primary human atrial myofibroblasts. Cell Death Dis.

[401] Rangwala R, Chang YC, Hu J, Algazy KM, Evans TL, Fecher LA (2014). Combined MTOR and autophagy inhibition: phase I trial of hydroxychloroquine and temsirolimus in patients with advanced solid tumors and melanoma. Autophagy.

[402] Rosenfeld MR, Ye X, Supko JG, Desideri S, Grossman SA, Brem S (2014). A phase I/II trial of hydroxychloroquine in conjunction with radiation therapy and concurrent and adjuvant temozolomide in patients with newly diagnosed glioblastoma multiforme. Autophagy.

[403] Karasic TB, Brown TJ, Schneider C, Teitelbaum UR, Reiss KA, Mitchell TC (2022). Phase I Trial of Regorafenib, Hydroxychloroquine, and Entinostat in Metastatic Colorectal Cancer. Oncologist.

[404] Roth GJ, Binder R, Colbatzky F, Dallinger C, Schlenker-Herceg R, Hilberg F (2015). Nintedanib: from discovery to the clinic. J Med Chem.

[405] Mosca E, Federa A, Pirker C, Schosserer M, Liendl L, Eckhard M (2024). The tyrosine kinase inhibitor Nintedanib induces lysosomal dysfunctionality: Role of protonation-dependent crystallization processes. Chem Biol Interact.

[406] Englinger B, Kallus S, Senkiv J, Heilos D, Gabler L, van Schoonhoven S (2017). Intrinsic fluorescence of the clinically approved multikinase inhibitor nintedanib reveals lysosomal sequestration as resistance mechanism in FGFR-driven lung cancer. J Exp Clin Cancer Res.

[407] Zhu H, Xia MM, Tong KH, Duan WB (2023). Nintedanib Induces the Autophagy-Dependent Death of Gastric Cancer Cells by Inhibiting the STAT3/Beclin1 Pathway. Dig Dis Sci.

[408] Luo L, Wang X, Liao YP, Xu X, Chang CH, Nel AE (2024). Reprogramming the pancreatic cancer stroma and immune landscape by a silicasome nanocarrier delivering nintedanib, a protein tyrosine kinase inhibitor. Nano Today.

[409] Gabasa M, Ikemori R, Hilberg F, Reguart N, Alcaraz J (2017). Nintedanib selectively inhibits the activation and tumour-promoting effects of fibroblasts from lung adenocarcinoma patients. Br J Cancer.

[410] Kato R, Haratani K, Hayashi H, Sakai K, Sakai H, Kawakami H (2021). Nintedanib promotes antitumour immunity and shows antitumour activity in combination with PD-1 blockade in mice: potential role of cancer-associated fibroblasts. Br J Cancer.

[411] Ghazipura M, Mammen MJ, Herman DD, Hon SM, Bissell BD, Macrea M (2022). Nintedanib in Progressive Pulmonary Fibrosis: A Systematic Review and Meta-Analysis. Ann Am Thorac Soc.

[412] Lamb YN (2021). Nintedanib: A Review in Fibrotic Interstitial Lung Diseases. Drugs.

[413] Nagy S, Denis O, Hussein A, Kesselman MM (2025). The Impact of Antihistamines on Immunotherapy: A Systematic Review. Cureus.

[414] Li L, Liu R, Peng C, Chen X, Li J (2022). Pharmacogenomics for the efficacy and side effects of antihistamines. Exp Dermatol.

